# Supplementary material for: Effects of Citric Acid, Synbiotic, and Probiotic Supplementation Through Drinking Water on Growth Performance, Carcass Yield, and Blood Biochemistry of Broiler Chickens
Source: Animals (Basel). 2025 Apr 18;15(8):1168. doi: 10.3390/ani15081168 (PMC12024077; doi:10.3390/ani15081168)
Supplement: Supplementary file 1 [file animals-15-01168-s001.zip › animals-3578156-supplementary.pdf]

COMPARISON OF CITRIC ACID, SYNBIOTIC AND PROBIOTIC THROUGH DRINKING WATER ON GROWTH PERFORMANCE, CARCASS YIELD AND BLOOD BIOCHEMICAL PARAMETERS IN BROILER

A THESIS

Submitted to the Department of Dairy And Poultry Science
Hajee Mohammad Danesh Science And Technology University, Dinajpur
In partial fulfillment of the requirement for the degree of

Master of Science
in
Poultry Science

By

MD. SHAHADOT HOSSAIN
Roll No. : 20230526
Registration No.: 43342
Session: 2014-2015

Department of Dairy And Poultry Science
Hajee Mohammad Danesh Science And Technology University
Dinajpur

January 2023

COMPARISON OF CITRIC ACID, SYNBIOTIC AND PROBIOTIC THROUGH DRINKING WATER ON GROWTH PERFORMANCE, CARCASS YIELD AND BLOOD BIOCHEMICAL PARAMETERS IN BROILER

A THESIS

Submitted to the Department of Dairy And Poultry Science
Hajee Mohammad Danesh Science And Technology Universuty, Dianjpur
In partial fulfillment of the requirement for the degree of

Master of Science
in
Poultry Science

BY

MD. SHAHADOT HOSSAIN

Approved as to style and content by

(……………………………)	(………………………)
Supervisor	Co-Supervisor

(…………………………………………..)
Chairman, Defense Committee
and
Head, Department of Dairy And Poultry Science
Hajee Mohammad Danesh Science And Technology University, Dinajpur

January 2023

ACKNOWLEDGEMENTS

In the name of Allah, the supreme authority of this universe

The author is constantly grateful to Almighty Allah for his never-ending blessing on the successful completion of the work. All thanks is given to Almighty Allah for His compassion and mercy in allowing the author to successfully complete this thesis for the degree of Masters of Science (MS) in Poultry Science.

The author wishes to express his heartfelt gratitude and gratitude to his beloved teacher and research supervisor, ***Professor Dr. ....*** *Department of Dairy and Poultry Science, Hajee Mohammad Sanesh Science And Technology University (HSTU), Dinajpur, for his cordial and scholastic guidance, continuous encouragement, valuable suggestions, and all-around assistance throughout the course of the research work and finalizing this manuscript.*

The author is ever grateful and immensely indebted to his honorable and respected teacher andresearch co-supervisor, ***Professor Dr.........................****, Department of Dairy and Poultry Science, Hajee Mohammad Sanesh Science And Technology University (HSTU), Dinajpur,, for his keen interest, valuable advice, kind cooperation and encouragement in completing the research work.*

*The author takes an opportunity to express his sincere appreciation and profound gratitude to****Professor*** *...............................and Head, Department of Dairy and Poultry Science, Hajee Mohammad Sanesh Science And Technology University (HSTU), Dinajpur, for his scholastic guidance, helpful advice, necessary instructions and constructive criticism during the course of work.*

The author extends his grateful acknowledgement to his all of the respected teachers for their generosity and suggestion, constructive comments and carrying out this research work at HSTU Poultry Farm.

The author feels happy to express his heartfelt gratitude to Naim, Hazamin, Shirin, Oishi and Nazrul vai for their kind help to successfully complete his research work.

The author expresses heartiest gratitude and gratefulness to his beloved parents A. Khalek Howlader and Lalmoti Begum, elder sister Rexona Parvin Mukta and Rozina Afrin Moni and all members of his family and relatives for their sacrifices, blessings and encouragement that pave the way of his higher education at Hajee Mohammad Sanesh Science And Technology University and made the dream true finally.

The Author

January 2023

COMPARISON OF CITRIC ACID, SYNBIOTIC AND PROBIOTIC THROUGH DRINKING WATER ON GROWTH PERFORMANCE, CARCASS YIELD AND BLOOD BIOCHEMICAL PARAMETERS IN BROILER

ABSTRACT

The experiment was conducted to compare the effect of citric acid, synbiotic and probiotic as alternative to antibiotic feed additives on growth performance in broiler. A total of 400 day old Cobb 500 as hatched broiler chicks were randomly assigned into 4 different treatment groups for five weeks. The treatment groups were; control (no additives), citric acid (2.5g/liter water), synbiotic (0.2g/liter water) and probiotic (0.5g/liter water). Each treatment consisted of four replicates and of each 25 chicks. Throughout the experiment body weight, body weight gain, feed intake and feed conversion ratio were recorded as growth performance. Carcass characteristics and blood biochemical profile were also taken after the end of feeding trial. The supplementation of additives in drinking water had significant ($P<0.05$) effect on growth performances. Results showed that probiotic treated broilers had significantly ($P<0.05$) higher body weight and body weight gain at the end of the experiment compared to control group. FCR was improved significantly ($P<0.05$) in probiotic group (1.62) compare to the control group (1.74), citric acid group (1.66) and synbiotic group (1.70). There were no significant difference in meat yield (breast meat, thigh meat, drumstick meat and wing meat), bone development (thigh bone, drumstick bone and wing bone) and carcass weight (head, neck, leg, liver, heart) among different treatment groups. Total cholesterol and LDL found significant ($P<0.05$) difference among the treated groups whereas TG and HDL were not differ significantly. With regards to profit, probiotic groups showed higher profitability compared to control group. Based on the results of this study suggest that the addition of citric acid, synbiotic, and probiotic to drinking water could be a potential additive in broiler and probiotic could be considered as the alternative to antibiotic in broiler production.

Keywords: Broiler, feed additives, growth performance, meat yield, blood parameters

CONTENTS

CHAPTER NO.	TITLE	PAGE NO.
	**ACKNOWLEDGEMENTS**	**iv**
	**ABSTRACT**	**v**
	**CONTENTS**	**vi-viii**
	**LIST OF TABLES**	**ix**
	**LIST OF FIGURES**	**x**
	**LIST OF APPENDICES**	**xi**
	**ABBREVIATIONS AND SYMBOLS**	**xii**
**1**	**INTRODUCTION**	**1-4**
**2**	**REVIEW OF LITERATURE**	**5-19**
	2.1 Alternatives to antibiotic growth promoters	5
	2.1.1 Citric acid	5
	2.1.1.1 Use of citric acid in poultry diet	6
	2.1.1.2 Effect of citric acid on growth performance of broilers	6
	2.1.1.3 Effect of citric acid on feed conversion	8
	2.1.1.4 Effect of citric acid on nutrient digestibility	9
	2.1.1.5 Effect of citric acid on gut microflora	10
	2.1.2 Synbiotic	10
	2.1.2.1 Effect of synbiotic on the performance of broiler	11
	2.1.3 Probiotics	13
	2.1.3.1 Use of probiotics in broiler diet	13
	2.1.3.2 Mode of action of probiotics	15
	2.1.3.3 Effect of probiotic on growth rate	15
	2.1.3.4 Effect of probiotics on feed intake	16
	2.1.3.5 Effects of probiotics on FCR	17
	2.2 Effect of additives on blood parameters	17
	2.3 Research gap	18

**3**	**MATERIALS AND METHODS**	**20-32**
	3.1 Statement of the experiment	20

CONTENTS (Contd.)

CHAPTER NO.	TITLE	PAGE NO.
	3.2 Collection of the experimental birds	20
	3.3 Preparation of the experimental house	20
	3.4 Experimental design	21
	3.5 Source of additives	21
	3.5.1 Collection of citric acid	21
	3.5.2 Collection of probiotic	21
	3.5.3 Collection of synbiotic	22
	3.6 Experimental diet	22
	3.7 Management of experimental birds	23
	3.7.1 Feed and water management	23
	3.7.2 Litter management	24
	3.7.3 Brooding of chicks	24
	3.7.4 Lighting management	25
	3.7.5 Vaccination	25
	3.8 Biosecurity	25
	3.9 Processing of broilers	26
	3.10 Data collection and record keeping	35
	3.10.1 Body weight	26
	3.10.2 Body weight gain	26
	3.10.3 Feed intake	26
	3.10.4 Feed conversion ratio (FCR)	27
	3.10.5 Temperature and humidity	27
	3.11 Blood collection	27
	3.12 Blood collection and separation of serum	28
	3.13 Serum biochemical parameters	28
	3.13.1 Determination of serum total cholesterol	28

	3.13.2 Determination of Triglycerides	30
	3.13.3 Determination of HDL	31
	3.13.4 Determination of LDL	31

CONTENTS (Contd.)

CHAPTER NO.	TITLE	PAGE NO.
	3.14 Cost-benefit analysis	32
	3.15 Statistical analysis	32
**4**	**RESULTS**	**33-40**
	4.1 Body weight and body weight gain	33
	4.2 Feed intake	35
	4.3 Feed conversion ratio	35
	4.4 Meat yield and bone development	37
	4.5 Dressing parameters of broiler	38
	4.6 Serum biochemical parameters	39
**5**	**DISCUSSION**	**41-46**
	5.1 Body weight and body weight gain	41
	5.2 Feed intake	42
	5.3 Feed conversion ratio	43
	5.4 Meat yield, bone development and different dressing parameters	44
	5.5 Serum biochemical parameters	45
	5.6 Cost effectiveness of production	46
**6**	**SUMMARY AND CONCLUSION**	**47-48**
	**REFERENCES**	**49-61**
	**APPENDICES**	**62-67**

LIST OF TABLES

TABLES NO.	TITLE	PAGE NO.
3.1	Layout of the experiment	21
3.2	Basic composition of probiotics	22
3.3	Basic composition of synbiotics	22
3.4	Ingredients and chemical composition of broiler starter and grower diets	23
3.5	Vaccination schedule	25
4.1	Growth performance of broiler in different dietary treatments at different age	33
4.2	Feed Intake of Broiler in different dietary treatments	35
4.3	Feed conversion ratio of broiler in different dietary treatments	36
4.4	Meat yield and bone development of broiler in different dietary treatments	37
4.5	Dressing parameters of broiler in different dietary treatments (% in relation to body weight)	38
4.6	Blood biochemical parameters of broiler in different dietary treatments (mg/dl)	39
4.7	Cost benefit analysis of broiler in different dietary treatments	40

LIST OF FIGURES

FIGURES NO.	TITLE	PAGE NO.
4.1	Final body weight of broiler in different treatments at different ages	34
4.2	Feed conversion ratio of broiler in different treatments at different ages	36

LIST OF APPENDICES

FIGURES NO.	TITLE	PAGE NO.
1	Body weight (g/bird) of broilers fed on different dietary treatments at different age	62
2	Body weight gain (g/bird) of broilers fed on different dietary treatments at different age	63
3	Feed intake (g/bird) of broilers fed on different dietary treatments at different age	64
4	FCR of broilers fed on different dietary treatments at different age	65
5	Blood biochemical parameters of broilers in different dietary treatments	66
6	Dressing parameters and meat and bone development of broilers fed on different dietary treatments (% in relation to body weight)	67

ABBREVIATIONS AND SYMBOLS

SYMBOLS		ABBREVIATIONS
%	=	Percent
/	=	Per
::	=	Ratio
@	=	At the rate of
+	=	Plus
°C	=	Celsius
ALP	=	Alkaline Phosphatase
BAU	=	Bangladesh Agricultural University
CF	=	Crude Fibre
Cm	=	Centimeter
et al.	=	Associates
FCR	=	Feed Conversion Ratio
g/b	=	Gram per broiler
g/kg	=	Gram per Kilogram
HDL	=	High Density Lipoprotein
IBD	=	Infectious Bursal Disease
Kcal	=	Kilo Calorie
ME	=	Metabolizable Energy
Meth	=	Methionine
ND	=	Newcastle Disease
NS	=	Nigella sativa
PUFA	=	Poly Unsaturated Fatty Acid
R	=	Replication
T	=	Treatment
TG	=	Triglyceride
Tk	=	Taka
Vitamin	=	Vitamin
WHO	=	World Health Organization
Wt.	=	Weight

CHAPTER 1

INTRODUCTION

Poultry sector has brought a revolutionary change in socio-economic condition by resolving the unemployment problem and providing high quality cheaper white protein all over the world. During the past two decades, this sector has been transformed from backyard rearing to commercial organized, scientific and potential industry in the world (Alkhalf *et al.*, 2010). With the commercialization, poultry producers utilize antibiotic growth promoters to improve feed consumption and growth performance. In Bangladesh, farmers are very much dependent on antibiotic growth promoters for their birds for enhancing better productivity. But, the indiscriminate use of antibiotics in livestock, specially poultry industry resulted many concerns; development of drug-resistant bacteria, drug residues in the body of the birds, and imbalance of normal microflora. As well as its adverse effects in poultry health and its residues in meat can make danger for human health (Furtula *et al.,* 2010). Recently, the European Union reported that antibiotic resistance causes 25,000 patients died each year and €2.5 million in medical healthcare costs (Ziggers, 2011). In India, over 58,000 babies died each year from infections caused by drug-resistant bacteria. Nevertheless, a number of antibiotics are still allowed for use at sub-therapeutic purposes in the USA. World Health Organization (WHO, 1997) has recommended antibiotics should be phased out from the poultry diet and replaced by alternatives which have no adverse effect on the consumer health (Bywater, 2005). Considering this, European Union (EU) already banned the use of antibiotics at sub-therapeutic levels in 2006. Therefore the use of antibiotic as growth promoters has been banned in many countries and these lead to investigations of alternative feed additives in animal production (Casewell *et al.,* 2003).

On the other hand, feed antibiotics, which have been used for promoting growth in farm animals, were shown to negatively affect profitability of the animal production. This situation therefore, propelled animal nutritionists and

researchers to search for other non-therapeutic alternatives for poultry such as citric acids (CA) (Panda *et al.,* 2009), probiotics, prebiotics, synbiotics, herbs and essential oils (Islam, 2012).

The use of citric acid in poultry production has been considered as safe therefore, they were allowed to be used as feed additive by the European Union (Adil *et al.,* 2011). Citric acids have made a great contribution to the profitability in the poultry production and also provided people with the healthy and nutritious poultry products. It also improves the solubility of the feed ingredients, digestion and absorption of the nutrients by modifying intestinal pH (Patten and Waldroup, 1988). Moreover, most of the research during last decade shows that citric acids are excellent promoters of growth performance and gut health in commercial poultry production (Sohail *et al.,* 2015). Therefore, it is important to realize and highlight their importance, impact and mode of action, to be able to maximize the benefits when included in poultry diets.

Citric acid is approved at European Union (EU) and it shows enough antimicrobial action to conserve the feed against bacterial spoilage, to reduce undesirable bacteria (e.g. *E. coli.*) in the gastrointestinal tract. The use of citric acid creates an acidic environment (pH 3.5 to 4.0) in the gut that favors the development of *lactobacilli* (Chowdhury *et al.,* 2009). It is one of the most significant compounds involved in the physiological oxidation of fats, proteins and carbohydrates to carbon dioxide and water. Citric acid increased the digestibility of protein and fibre (Atapattu and Nelligaswatta, 2005), improved live weight gain, feed conversion efficiency, absorption of minerals (Chowdhury *et al*., 2009; Shen *et al.,* 2005; Moghadam *et al.,* 2006; Nezhad *et al.,* 2007), it reduces the available P requirement (Boling *et al*., 2000). It decreases feed intake and increased average daily live weight gain, using lover feed per kg body weight gain of broiler. It also decreases pH of caecal digesta (Jozefiak and Rutkowski, 2005), crop and gizzard (Andrys *et al*., 2003) and intestine (Denil *et al*., 2003; Rahmani *et al.,* 2011) in broiler chicks. It reduced microbial load (Gunal *et al.,* 2006; Ivanov, 2005) has better immune response in broilers

(Abdel-Fattah *et al.*, 2008; Rahmani *et al.,* 2011). The citric acids had better immune response and disease resistance as indicated by higher serum globulin and weight and height of lymphoid organs which have different immunological advances and acting as a natural barrier against pathogenic bacteria and toxic substances (Paul *et al.,* 2007). Considering these facts citric acid was safe for human and can be used as growth promoter in broiler production up to 0.5% level in ration (Chowdhury *et al.,* 2009).

Probiotics are defined as feed additives that contain live microorganisms and promote beneficial effects to the host by favoring the balance of the intestinal microbiota (Fuller, 1989). Probiotics are non-digestible feed ingredients that can positively affect the animal organism by stimulating the activity and growth of beneficial native bacteria in the gastrointestinal tract and eliminate the pathogenic ones. It has been suggested that probiotics are convincing alternative for antibiotics as therapeutic and growth promoting agent (Cavazzoni *et al*, 1998; Martins *et al.,* 2005). It improves the productive and economic efficiency of broiler chicks. This could be because it improves feed conversion efficiency and lowers feed costs, which is the most important component of the poultry business. Probiotics are dietary supplement that increase the population of microflora in the intestinal tract, which is necessary for proper food preparation, strengthens the immune system and helps bird to digest their food more quickly, allowing them to stay healthy and gain weight faster. Probiotic efficacy may depend on factors such as microbial species composition (e.g., single or multi-strain) and viability, administration level, application method, frequency of application, overall diet, bird age, overall farm hygiene and environmental stress factors. It is also used as feed additive and it has a good impact on the performance of poultry (Stavric and Kornegay, 2008).

Synbiotics, a combination of probiotics and prebiotics have been introduced as an alternative to antibiotics and growth promoters in poultry industry. It has a synergistic effect that can improve the health status of broiler, the efficiency of feed ingredients, antibacterial activity, immunity to infection and the

performance of broiler chickens. The combination of a prebiotic and probiotic in one product has been shown to delegate benefits beyond those of either and it may be the combination of synbiotics that beneficially affected the host by improving the survival. Synbiotic bacteria were demonstrated to increase intestinal morphology and nutritional absorption in broiler chickens, resulting in better performance (Hassanpour *et al*., 2013). Only a few studies have reported on the most effective technique to employ synbiotic in poultry (Li *et al*., 2020). Finding the optimal probiotic and prebiotic combination, as well as analyzing their synergistic effects for usage as prospective synbiotics to maintain appropriate health, necessitates a great deal of effort. Madej *etal*., (2015) discovered that an in vivo injection of inulin (prebiotic) mixed with *lactobacillus* bacteria changed the development of many immune organs in broilers. Al-Sultan *et al*., (2016) discovered that, when compared to prebiotics and organic acids, the synbiotic supplemented group had the greatest final body weight and time-dependent weight growth, followed by the probiotic-supplemented group. FCR is higher, and the antibody response to Newcastle disease is stronger.

With this in mind, the following objectives for the present research project were established:

1. To evaluate the growth performance of broilers on different additives through drinking water.
2. To compare among different antibiotic alternatives for the production of safe broiler meat.
3. To evaluate meat yield characteristics of broilers on different antibiotic alternatives.

CHAPTER 2

REVIEW OF LTIERATURE

This "Review of Literature" chapter is organized with a view to review some previous research relevant to thepresent study. Scientists performed various surveys and research works on citric acids, synbiotics and probiotics in numerous countries. For gathering genuine evidence and major information the review of literature associated with research title has been conveniently brought under the following sub-headings:

2.1 Alternatives to antibiotic growth promoters

The preceding sections demonstrate the negative consequences of antibiotic growthpromoters (AGPs), particularly on human health. Given the negative effects of AGPs and the EU's removal of AGPs, scientists are actively seeking a good replacementto AGPs. Probiotics, citric acids, synbiotics have recently been proposed as alternatives to antibiotics (Patterson and Burkholder, 2003).

2.1.1 Citric acid

Citric acid originates naturally and acts as non-therapeutic additives. More than a million tons of citric acid is manufactured every year. It is used widely as an acidifier, as a flavoring and chelating agent (Apleblat, 2014). It also used as alternative to antibiotic in broiler diet. Citric acid was first isolated in 1784 by the chemist Carl Wilhelm Scheele, who crystallized it from juice. Citric acid exists in greater than trace amounts in an exceedingly type of variety of fruits and vegetables, most notably citrus fruits. In 1893, C. Wehmer discovered *Penicillium* mold could produce acid from sugar. Citric acid enhance bird's immunity, improves response to vaccination, balances guts microflora, controls diarrhea and increases productivity and decrease mortality.

2.1.1.1 Use of citric acid in poultry diet

The citric acid and probiotics are the most hopeful alternatives for poultry (Gunal *et al.,* 2006). Their supplementation within the diet of broilers enhanced nutrient utilization, growth, and feed efficiency (Jin *et al*., 1998; Denil *et al.,* 2003). Citric acid is approved as a preservative to protect cereals and other feedstuff against microbial decomposition but without minimum and maximum level for inclusion. The use of citric acid creates an acidic environment (pH 3.5 to 4.0) in the gut that favors the development of lactobacilli and inhibits the replication of *Escherichia coli*, Salmonella, and other gram-negative bacteria. It shows enough antimicrobial action to preserve against bacterial spoilage, but simultaneously reduces pathogenic bacteria in the gastrointestinal tract (example *E. coli.)* and ultimately improves growth rate of different animal species (Eidelburger and Kirchgaessner, 1994; Falkowski and Aherne, 1984) at an extent comparable to antimicrobial growth promoters.

2.1.1.2 Effect of citric acid on growth performance of broilers

Citric acid has growth-promoting properties and can be used as alternatives to antibiotics (Fascina *et al*., 2012). Recently, Brzoska *et al.,* (2013) reported that citric acid (0.3–0.9%) had a growth enhancing and mortality-reducing effect in broiler chickens, with no significant influence on carcass yield or proportion of individual carcass parts. Islam *et al.,* (2012) estimated that CA up to a level of 1.25% of the diet increased performance, carcass weight and carcass quality and reported that the addition of 0.75% CA in a standard diet is suitable for broiler growth. Several authors investigated the effect of doses of citric acid on growth performance.

Abdel Fattah *et al.,* (2008) demonstrated that, addition of citric acid improved the live body weight and body weight gain of broiler.

Chowdhury *et al.,* (2009) reported that citric acid significantly ($p<0.05$) increased the live weight and weight gain of broiler.

Nezhad *et al.,* (2007) reported that supplementation of CA (0.0, 2.5%) with a maize soybean meal based diet improved the live weight gain of broilers and the effects of the interaction between CA and microbial phytase showed the significant improvement on live weight gain.

Moghadam *et al.,* (2006) observed that significant result on interactive effects of CA and phosphorus on 21 days body weight of broilers. The addition of phytase with CA, ascorbic acid and vitamin D3 to the low Ca diet in broilers improved the body weight by 18% (Afsharmanesh and Pourreza, 2005) but insignificant growth performance of broilers fed CA (1 and 2%) with rice by-product based diets (Atapattu and Nelligaswatta, 2005). However, the result on the beneficial effect of CA on weight gain reported by many researchers.

Ivanov (2005) reported that lactic acid bacteria, baker's yeast and CA use in broiler diet the average daily weight gain was 57g in treated chicks whereas, the body weight of control chicks was 26.5% lower than treated chicks.

Zhang *et al.,* (2005) carried out an experiment by feeding fumeric acid, citric acid and malic acid together. They reported that the organic acid mixture had better body weight gain, feed consumption, FCR and survivability.

Shen-HuiFang *et al.,* (2005) investigated the same effect of the levels (0.3%, 0.5% and 0.7%) of CA. Citric acid in combination with other organic acids or other products also improved the performance of broiler. During this connection, the mixture of organic acids namely fumaric, lactic, citric and ascorbic acids improved the broilers performance, even within the absence of antibiotic, an effects being observed with reference to the intestinal morphology and lipid utilization (Maiorka *et al.*, 2004). Muzaffar *et al.,* (2003) studied the effect of organic acid along with probiotic. They reported that organic acid group showed better weight gain, feed efficiency, FCR and good carcass quality than control and others.

The above information's make it clear that CA alone a combined with others

have positive effect on growth performance of broiler.

2.1.1.3 Effect of citric acid on feed conversion

Dietary addition of citric acid improved feed conversion of broiler chicks as compared to those of un-supplemented diet reported by Abdel- Fattah et al., (2008).

Isabel and Santos (2009); Parker (2011), who investigated that organic acid has significantly affected the feed conversion ratio (FCR).

Nezhad *et al.,* (2007) reported that, feed conversion efficiency improved on diets supplemented with three levels of CA (0.0, 2.5 and 5.0%) and the effect of interaction between CA and microbial phytase was significant on feed conversion efficiency in broilers.

Moghadam *et al.,* (2006) reported that insignificant effects of CA (0, 1.5 and 3.0%) on feed conversion in broilers.

Shen-Huifang *et al.,* (2005) demonstrated best feed conversion ratio with addition of 0.3% CA in chicken. Organic acid (CA and ascorbic acid) in combination with microbial phytase and vitamin D3 showed better feed conversion ratio (FCR) when supplemented to broilers at low-phosphorus and low-calcium diet (Afsharmanesh and Pourreza, 2005). Feed conversion ratio was not significantly affected by the inclusion of two levels of CA (1% and 2%) in broiler chickens fed rice by-product based diet showed insignificant effect on feed conversion ratio (Atapattu and Nelligaswatta, 2005). Broiler diet with organic acid and probiotic supplemented group shown better feed conversion efficiency than control and supplemented group (Gunal *et al.*, 2006).

Andrys *et al.,* (2003) observed insignificant differences in term of feed conversion between controls and FA (phosphoric acid and CA) treated groups of broilers.

Celik *et al.*, (2003) found better feed conversion in broiler supplementing acidifier diet. Hassan *et al.*, (2010) stated that organic acids significantly ($p<0.001$) improved feed conversion ratio. The feed conversion ratio results in present study are supported by Samanta *et al.,* (2010) who stated that organic acids reduce E. coli and other harmful bacteria which increased poultry growth.

Boling *et al.,* (2000) reported that organic acid produces acidic condition that makes the nutrients more available for better performance.

2.1.1.4 Effect of citric acid on nutrient digestibility

Citric acid normally used as an acidifier in poultry feeds have been considered to be attractive alternatives for improving nutrient digestibility. Citric acid may weaken the structure of crude fiber thus making crude protein and phytate associated with them more susceptible for enzymatic digestion and facilitate the nutrient digestibility. Dietary acidification increases gastric proteolysis and protein, amino acid digestibility.

Ghazala *et al.,* (2008) reported that dietary 0. 2% citric acid improved both ME and nutrient digestibility and increased the retention of DM, CP and neutral detergent fibre (Ao *et al*. 2009).

Nourmohammadi *et al.,* (2012) reported that inclusion of 1000 U/kg MP and 3% CA in grower and finisher diets significantly increased the digestibility and growth performance in broiler chickens. In addition, by modifying intestinal pH, organic acids also improve the solubility of the feed ingredients, digestion and absorption of the nutrients.

Samanta *et al.,* (2010) reported that citric acids raised gastric proteolysis and improved the digestibility of protein and amino acids. Dietary addition of organic acids can also improve the digestibility of minerals and increase the utilization of the phytate phosphorus (P) (Bioling *et al.,* 2000).

Emami et al., (2013) reported that broilers fed the control diet (without microbial phytase enzyme and organic acid) had the lowest CP and EE digestibility (0.7751 and 0.7949, respectively), which were improved ($P<0.001$ and $P=0.010$, respectively) by the addition of Phytase + organic acid (0.8858 and 0.8561, respectively) to the control diet. Organic acid supplemented group had longer and thicker villi than that of controls and consequently, better efficiency in digestion and absorption of feed (Abdel Fattah *et al.,* 2008).

Gong-YiGeng *et al.,* (2006) reported that, under supplementation of bacterial phytase or CA in broiler, crude protein (CP) utilization was lower than that of control.

Afsharmanesh and Pourreza (2005) stated that, addition of CA and ascorbic acid to low Ca diets increased protein digestibility by 23% and Vargas-Rodriguez *et al.,* (2002) also reported that when 3 levels of citric acid (0.0, 0.6 and 2%) used in diet of laying hens then N in excreta decreased.

2.1.1.5 Effect of citric acid on gut microflora

Acidifiers are believed to promote normal gut flora, enhanced gut integrity, improved performance and productivity. Citric acids are particularly effective against acid-intolerant species such as E. coli, Salmonella and Campylobacter have been used through feed by different researchers (Izat *et al.,* 1990; Luckstadt, 2007; Thompson and Hinton, 1997). The incorporation of citric acid specialy at 4.5 and 6% inclusion levels into drinking water significantly declined bacillus, clostridium, coliform, facultative aerobic and other bacteria of gizzard, ceca, and feces compared with the control (Hassan MA *et al.,* 2010).

2.1.2 Synbiotic

In its most basic form, a synbiotic is a mix of probiotics and prebiotics (Collins and Gibson, 1999). According to recent research, synbiotic products

boosted immunological condition in broiler chicks (Zhang *et al.,* 2006). According to (Awad *et al.,* 2008) research, synbiotics can improve glucose absorption in chickens. A synthetic substance with the same potential to boost broiler performance as avilamycin (an antibiotic growth promoter) was tested (Mohnl *et al.,* 2007).

Liong and Shah (2006) concluded that the use of synbiotics in broilers modulates the concentration of organic acids and lowers cholesterol levels.

2.1.2.1 Effect of synbiotic on the performance of broilers

As observed in various studies and reviews, the combination supplementation of poultry diets with probiotics and prebiotics (synbiotic) is more beneficial than a single supplementation and in some circumstances, even congruent with antibiotic treatments (Gaggia *et al*., 2010, Tayeri *et al*., 2018). Improvements in feed efficiency in broiler chickens have been linked to synbiotic supplementation's potential modulatory influence on gastro-intestinal bacteria colonization (Brugaletta *et al.,* 2020).

Nisar *et al*., (2020) conduct an experiment aimed to explore the effect of dietary supplementation of synbiotics on growth performance, carcass characteristics and nutrient digestibility in broiler chicken. They employ 300 day-old cobb-500 broiler chicks, which are randomly assigned to one of five dietary treatment groups. Each treatment featured six replicates, each of which contained ten chicks. The experimental diets were labeled A, B, C, D, and E, with A being the control diet, and supplemented with 0, 700, 1200, 1700, or 2200 g/ton of feed synbiotics. When compared to the control, group C's feed consumption was reduced. All treatments had equal body weight. However, as compared to other nutritional treatments, group C had a much higher feed conversion ratio. Nutrient digestibility was higher in groups B and C compared to the control. Carcass properties were unaffected and remained constant throughout all treatments.

Fornazier *et al.*, (2019) use 640 male chicks (Cobb 500) to conduct an experiment to examine the effects of a symbiosis on 1–42-d-old broiler chicks' performance, and carcass yield. The birds were randomly assigned to one of four treatments, each containing eight replicates (pens) of 20 birds. Different quantities of symbiotic (probiotic bacteria, exogenous enzymes, and autolyzed yeast) in the meals were used in the treatments: 0.0, 0.5, 1.0, and 1.5 kg/t. They found that synbiotic improves animal performance, particularly weight gain, which increased by 4.3 percent in the treatment with 1.5 kg/t inclusion compared to the diet without synbiotic. They also reported that inclusion of the symbiosis in the meals improved carcass yield and breast yield.

The research is carried out by Tayeri *et al.,* (2018) where the effects of antibiotics, probiotics, synbiotics, and prebiotics on broiler performance and carcass parameters were compared. They state that the synbiotic therapy lowered the feed conversion ratio when compared to the control and antibiotic treatments, and that the gizzard and spleen weights were comparatively higher. They conclude that using prebiotics, probiotics, and synbiotics instead of antibiotics has considerable performance and health benefits for broilers.

Raksasiri *et al.*, (2018) studied the effect of synbiotic in broiler diets on productive performance, intestinal histomorphology and carcass quality. The researchers discovered that feed intake differed dramatically with the addition of synbiotics at 0.05% of DM. Higher levels of synbiotic use resulted in lower feed conversion rates in all groups receiving synbiotic supplementation. Furthermore, nutritional treatment with 0.05% of DM synbiotic considerably boosted villus height. Furthermore, it was shown that with synbiotic 0.05% of DM of chicken feed supplementation of synbioticswere not effect on crypt depth, lactic acid bacteria, carcass quality, and meat quality.

Mousavi *et al.*, (2015) found that body weight increase (BW) in broilers fed

synbiotic diets was considerably greater than in birds fed control diets, especially at 0–2 weeksof age. They also reported that broiler consumption of metabolizable energy (ME) and crude protein (CP) increased when the synbiotic was fed at 0.075 and 0.15 % of the diet. Furthermore, while only birds given 0.075 % synbiotic had a better production index and revenue margin, live weight and feed efficiency were greater in broilers fed starter diets containing 0.1, 0.15, and 0.125 percent synbiotic when compared to the control diet.

The effect of in ovo injection of prebiotics and synbiotics on broiler chicken growth performance and meat quality attributes was investigated (Maiorano *et al.*, 2012), and providing synbiotics had a minimal influence on the performance parameters.

According to Awad *et al.*, (2008), using the synbiotic in broiler diets dramatically boosted body weight and growth, carcass yield percentage, and feed efficiency. Ghasemi *et al.*, (2010), on the other hand, found a substantial increase in body weight growth and an improvement in feed conversion ratio in broilers fed the synbiotic Biomin®IMBO at 0.1 and 0.15 % of diet from day-old to 42 days of age. They also found a substantial reduction in coccidian oocysts in broilers fed 0.15 % synbiotic diets compared to controls.

2.1.3 Probiotics

The term “probiotics” was first applied by Lilly and Stillwell (1965) to designate unknown growth promoting substances produced by a ciliate protozoan that stimulated the expansion of another ciliate. The term now covers a far broader group of organisms. Probiotics were defined by Parker (1974) as “organisms and substances which contribute to intestinal microbial balance” thus including both living organisms and non-living substances. Fuller (1989) criticized the use of the word 'substances' and described probiotics as "a live microbial feed supplement that optimizes the host animal's intestinal microbial balance". The

joint Food and Agriculture Organization of the United Nations (FAO) and World Health Organization (WHO) Working party defined probiotics as "live micro-organisms which when administered in adequate amounts confer a health benefit on the host" (FAO/ WHO, 2001). This definition is widely accepted and adopted by the International Scientific Association for Probiotics and Prebiotics (Hill *et al.,* 2014). Probiotics as a feed additive have been acclaimed to be a safe growth promoter in animals (Bansal *et al.,* 2011).

2.1.3.1 Use of probiotics in broiler diet

The use of probiotics is a viable option for improving poultry productivity and health. Probiotics are living bacteria that can be fed to chickens to improve their health. It may also lead to increased nutrient digestion and absorption and a more balanced gut flora (Wondwesen *et al.,* 2017).

Probiotics must be added to chicken production to stimulate hunger, promote intestinal microbial balance, stimulate the immune system, decrease pH, improve egg and meat quality and increase feed conversion ratio (Wondwesen *et al.,* 2017).

Pourakbari *et al.*, (2014) experimented on 201, day old male chickens which were allocated to one of five treatments (four replicates of 10 birds per treatment): control, and the same control diet supplemented with 0.005%, 0.01%, 0.015% and 0.02% probiotics. Probiotics in feed at 0.01% or higher levels of supplementation improved body weight gain (+12%) and feed conversion rate (-5%) compared with the control. There have been no effects on carcass traits, but the relative weights of drumsticks and wings showed increasing and decreasing linear responses, respectively, to probiotic supplementation level. Blood plasma glucose and albumin contents linearly increased (from 167.1 to 200.5 mg dl-1, and from 1.70 to 3.25 g dl-1) with increasing probiotic supplementation. Plasma glucose and albumin contents were lower in probiotic supplemented treatments (average contents 71.3 and 125.3 mg dl-1 vs. 92.6 and

149.9 mg dl-1 in the control).

Mansoub *et al., (*2011) reported that the effects of yogurt and probiotic on performance and serum composition of broiler chickens. As compared to the control group with the other groups observably to give improve performance in all of the experimental ($P<0.05$). According to the results, total cholesterol (Cholesterol), triglyceride (TG), HDL and LDL were measured in blood samples of day 42. The amount of total Cholesterol and triglyceride (TG) in the serum were showed a significant difference, but HDL was not significantly different among groups.

According to Kabir (2009), scientists are now focusing their efforts on establishing the delicate symbiotic link between chicken and their bacteria, particularly in the digestive tract, where they are important to both human and poultry health. Probiotics have a lot of potentiality to replace antibiotics because they don't cause germ resistance to grow and spread. Probiotics can be used as nutritional agents in chicken diets to promote growth, modulate intestinal microbiota and pathogen inhibition, immunomodulation and improve meat quality. According to (Midilli *et al.,* 2008) research, adding probiotics to the chicken diet improves the feed conversion ratio. This could be attributable to the fact that it improves the gut flora in birds. Probiotics improve the economic and productive efficiency of poultry farms and they also account for a tiny fraction of total or variable poultry production costs (Omar, 2014). This may be increase the feed conversion efficiency which reduces the cost of feed and increase the productivity of the farm (egg and meat produced per bird).

According to G.S. Ghadban (2002), probiotics competitively exclude the salmonella bacteria from the intestinal tract of the treated chicken. This means the number of antibodies in the bird's body will increase. Probiotics could be used as a non-antibiotic growth promoter feed additives in poultry production (Ashayerizadeh, 2011). According to the research report, probiotics improve the performance of all animal species (Ezema, 2013). The

introduction of probiotics in poultry feed is used to prevent bacterial infections (Kabir, 2009). Probiotics could also be used as vaccine in the poultry farm especially for the broiler production. According to the report of Irshad (2006), probiotics could substitute antibiotics in the poultry farm. It might reduce the negative impact of the antibiotics in the poultry product and public health issues.

Mastbaum *et al.,* (1997) used probiotic via drinking water in broilers. They reported that administration of probiotics via drinking water significantly affected live weight gain and feed conversion efficiency at the end of the day 41. They also emphasised that this beneficial effect was clearer at the end of the day 31.

2.1.3.2 Mode of action of probiotics

Different probiotics exert their effects through various mechanisms. The host birds benefit from probiotic bacteria, which create favorable conditions by preventing dangerous and pathogenic species in a variety of ways.

2.1.3.3 Effects of probiotics on growth rate

Probiotics can improve broiler chicken growth rates (Zhang and Kim, 2014). Probiotics staring from non-spore forming LAB to spore formers and yeast are evaluated for their potential to increase growth rates in commercial poultry production (Shim *et al.,* 2012).

Cao et al., (2013) found that E. faecium (HJEF005) at 109 cfu/kg of feed improved rate in male Cobb broilers challenged with E. coli, while Zhao et al., (2013) employing a different strain (LAB 12 – CGMCC 4847), fed at the speed of 2 ×109 cfu/kg of feed to male Ross broilers, found no growth effect.

According to Torshizi *et al.,* (2010), Generally the probiotic via drinking water increased body weight gain of broilers throughout production

periods compared to the control group ($p<0.05$). Provision of probiotic in water increased body weight gain compared to probiotic in the feed ($p<0.05$). The probiotic increased feed intake of birds during the grower phase and over the whole period compared to control ($p<0.01$). Probiotic increased daily body weight gain and feed intake of birds (Rahimi *et al.,* 2011).

2.1.3.4 Effect of probiotics on feed intake

Amerah *et al.,* (2013) administered a commercial probiotic (Enviva Pro 202 GT; Danisco Animal Nutrition, Marlborough, UK) containing three strains of *Bacillus subtilis* (strains BS8, 15AP4 and 2084) during grower/finisher phase of a 42-day feeding trial and found a reduction in feed intake of 2% along with reduction in FCR of 2.7%.

Fathi *et al.,* (2016) stated that insignificant decreased feed intake ($p<0.08$) was observed in both groups fed a diet supplemented with probiotics 200 and 400 g/t feed containing 4.109cfu/g of *Bacillus subtilis* compared to group receiving the control diet.

Saadia and Nagla (2010) reported feed intake values of different treated groups were approximately similar and lacked significance with layer flock that fed with *Saccharomyces cerevisiae*.

2.1.3.5 Effects of probiotics on FCR

Zhang *et al.,* (2021) showed that the supplementation of probiotics to daily drinking water had no obvious effect on average daily gain (ADG), average daily feed intake (ADFI) and FCR of both male and female chickens in their early growth period (0-21 days). From day 22 to 42 days, the treatment of probiotic efficiently elevated the BW, ADG and ADFI in female chickens when compared with the control, whereas no impact was observed on the FCR. In the probiotic group, the ADFI and FCR of male chickens significantly reduced, while body weight (BW) significantly increased. During the 0-42 days, probiotic supplementation markedly increased BW,

ADG and ADFI in female chickens and significantly improved BW and FCR in male chickens.

Fathi *et al.,* (2018) showed that there were no significant differences (*Bacillus subtilis*) in FCR between hens fed probiotic supplemented diets and hens fed the control diet.

Raka *et al.,* (2014) FCR stated highest result on control (2.05) and lowest on 0.30 % (v/v) liquid probiotics mixed culture supplementation (1.92) which was a better indication of improved FCR.

Abdelqader *et al.,* (2013) stated significant improvement in the feed efficiency or FCR value of *Bacillus subtilis* containing diet.

2.2 Effect of additives on blood parameters

Ashayerizadeh *et al.,* (2011) reported that probiotic, prebiotic, citric acid and synbiotic supplementation to the Ross 308 broiler diet at 42 days, only synbiotic highly significantly decreased serum cholesterol compared with the control being 3.71, 3.77, 3.58 and 4.15mmol/L, respectively. While, 42 Capcarova *et al.* (2010) reported that broilers was administrated with two type of the probiotics with concentration of 1×109 cfu of Lb.fermentum CCM 7158 and 2×109cfu of E.faecium M 74 in 1 g of nutrient medium in drinking water for 42 days, there was no significant effect in serum cholesterol compared with the control being 4.813, 4.862 and 4.428 mmol/L, respectively.

Mansoub *et al., (*2011) reported that the effects of yogurt and probiotic on performance and serum composition of broiler chickens. As compared to the control group with the other groups observably to give improve performance in all of the experimental (P<0.05). According to the results, total cholesterol (Cholesterol), triglyceride (TG), HDL and LDL were measured in blood samples of day 42. The amount of total Cholesterol and triglyceride (TG) in the serum were showed a significant difference, but HDL was not significantly different among groups.

Torshizi *et al.* (2010), Cholesterol and triglyceride concentrations in plasma were significantly reduced by probiotic application. The route of administration was not significant, although the water group showed lower plasma cholesterol in comparison to the feed group. Probiotic administration reduced plasma cholesterol and triglyceride concentrations.

2.3 Research gap

In broiler production, citric acid, probiotics and synbiotic are commonly employed as antibiotic alternatives. All three of these factors improve broiler performance and carcass yield. A great deal of work has been performed by scientists across the world investigating the effects of probiotic, synbiotic and organic acid on poultry production. More research works will be needed using these additives in poultry feed, since the composition of such useful feed additive varies from one to another focusing on body weight, growth rate and immunological status of broiler birds. Limited works have been done on comparing which strain is more effective either single or their combinations. Similarly, the performance of these additives may vary depending upon the strain, climatic conditions, their concentration, environmental stresses and so many factors may also be associated with the efficiency of these additives. Moreover, there is no recommendation for exact doses and length of feeding as well.

CHAPTER 3

MATERIALS AND METHODS

3.1 Statement of the experiment

This study was conducted with 400, day-old as hatched Cobb 500 broiler chicks in the Poultry Farm, Hajee Mohammad Danseh Science And Technology University (HSTU), Dinajpur to find out the effect of probiotic, synbiotic and citric acid on growth performance, biochemical parameters, mortality and economic appraisal of broilers. Duration of the feeding trial was 35 days.

3.2 Collection of the experimental birds

A total of 400 day-old Cobb 500 straight run commercial broiler chicks were used for this feeding trail and chicks were collected from Nourish Poultry and Hatchery Ltd. in Dinajpur Sadar, Dinajpur.

3.3 Preparation of the experimental house

The experiment was conducted in a semi-monitor type open-sided house. Wire net was used to divide the shed into 16 similar size pens each of 30 square feet (10 feet x 3 feet). There were three sections to the shed. The central section served as a walkway, while the remaining two sections housed experimental birds. The experimental room was brushed, swiped, cleaned and thoroughly washed with water. After that, bleaching powder was spread over the floor at a rate of 1kg/500 square feet and left undisturbed for 24 hours. The room was then disinfected using a 1% TH4+ solution (0.1 liter diluted solution per square foot), made by Sogeval in France and distributed by Century Agro Ltd in Bangladesh. Feeders, drinkers, buckets, and any other essential equipment were cleaned and disinfected with a 0.5 percent TH4+ solution as well.

3.4 Experimental layout

The experimental broiler chicks were split randomly into 4 dietary groups, with each group being replicated into 4 sub-groups. Each dietary group is made up of 100 chicks divided into 4 replicated pens, each with 25 chicks.

Control group = without feed additives

Citric acid group = 2.5g/liter of water

Synbiotic group = 0.2g/liter of water

Probiotic group = 0.5g/liter of water

The layout of the experiment is shown in Table 3.1.

Table 3.1 Layout of the experiment

Treatments	Birds per replication				Total
	R1	R2	R3	R4	
Control	25	25	25	25	100
Citric acid	25	25	25	25	100
Synbiotic	25	25	25	25	100
Probiotic	25	25	25	25	100
**Total**					400

3.5 Source of additives

3.5.1 Collection of citric acid

Citric acid was collected from the local market of Dinajpur town manufactured by Merck Specialities Private Limited, Warli, Mumbai. A dose of 2.5g/liter of citric acid was mixed with water.

3.5.2 Collection of probiotic

The trade name of the probiotic product used in the experiment was "Promax" manufactured by Indian Company named "Sanzyme Biologics (p) Ltd. and marked in Bangladesh by "Eskayef pharmaceuticals Ltd (SK+F)". According to manufacturer's instruction the inclusion rate of the product for commercial broiler was 0.5g/liter water.

Table 3.2: Basic component of probiotics (promax)

Component	Amount
Bacillus Subtilis	≥ 4.5×10^9cfu/g
Bacillus coagulans	≥ 4.5×10^9cfu/g
Saccharomyces boulardii	≥4.5×10^9cfu/g

3.5.3 Collection of synbiotic

A commercial synbiotic named "SynBac" was employed in the feeding trail. It was made in Thailand by K.M.P.BIOTECH CO., LTD. and distributed by PVF Agro Limited in Dhaka, Bangladesh.

Table 3.3: Basic component of Synbiotic (SynBac)

Component	Amount
Bacillus Subtilis	≥ 5×10^9cfu/g
Pediococcus acidilacticii	≥ 5×10^9cfu/g
Enterococcus faecium	≥ 5×10^9cfu/g
Saccharomyces cerevisiae	≥ 1×10^9cfu/g
Xylo-oligosaccharide	35g

3.6 Experimental diet

The broiler chicks were fed with standard broiler starter and broiler grower diets according to their requirements (Table-3.4). The starter diet was given to the birds from day-old to 14 days and then the grower diet was given from day 15 to the end of feeding trial (35 days). Commercial ready feed (Nourish Poultry Feed) was used as a starter diet and hand mixed feed was used as a grower diet. The required amount of probiotics, synbiotics and citric acids was weighted treatment-wise and it was then mixed with drinking water for each replication separately.

Table 3.4: Ingredients and chemical composition of broiler starter and grower diets

Ingredients (kg/100kg)	*Starter diet(0-14 days)	Grower diet(15-35days)

Maize	50.32	53
Rice polish	8.00	10.0
Soybean	29.00	22.50
Protein concentrate (CP, 60%)	8.00	8.00
Oyster shell	1.00	1.00
Salt	0.30	0.25
DL-Methionine (99%)	0.20	0.18
Lysine (98.5%)	0.03	0.03
Vitamin-mineral premix**	0.25	0.25
Soybean oil	3.50	4.00
Di calcium phosphate	0.25	0.25
Choline chloride (50%)	0.10	0.10
Total	100	100
**Chemical composition (calculated)**		
ME Kcal/kg	3050	3150
CP%	23.66	20.59
DM %	89.68	87.09
Lys %	1.05	1.06
Met %	0.65	0.63
Met + Cys %	1.00	0.92
Ca%	0.95	1.05
Available P %	0.45	0.42

***Composition of fresh ingredients was collected from Nourish poultry and hatchery Ltd.,Bangladesh (Islam *et al.* 2019, Paul *et al.* 2010)**

*****Vitamin-mineral premix supplied the following per kg of diet: Vit A, 12,000 IU; Vit D3, 4,000 IU; Vit E, 45 mg; Vit K3, 2.2 mg; Vit B1, 1.2 mg; Vit B2, 5.5 mg; Vit B6, 3 mg; Vit B12, 0.03 mg; Niacin, 50 mg; Panthothenic acid, 10 mg; Folic acid, 0.5 mg; Biotin, 0.08 mg, Mn, 70 mg; Fe, 48 mg; Cu, 5 mg; Zn, 60 mg; Se, 0.2 mg; I, 1 mg.***

3.7 Management of experimental birds

The following management procedures were performed throughout the experimental period and these management practices were identical for all treatment groups.

3.7.1 Feed and water management

Since, the DOC's were brought to the experimental site after a long journey, 5% glucose solution and vitamin-C were supplied to the chicks for the first 5 hours. For the first 4 days, feeds were given on newspaper and then on small trays. After 7 days small feeders along with trays were provided to adjust the chicks with the feeders. After placing the chicks in pens, two feeders and one drinker with a capacity of 7-liter water were provided in each pen, so that the birds can accustomed with feeders and drinkers. Feeders were cleaned in every week and drinkers were cleaned twice daily. Starter diet was provided for the first 2 weeks and grower diet was provided to the broiler up to 5th week of age. Feed was supplied three times daily, once in the morning, noon and afternoon in such a way that feeder was not kept empty. Citric acid was added to drinking water at a level of 2.5g/ liter , Synbiotics (SynBac) was added to drinking water at a level of 0.2g/ liter and 0.5gm probiotic (Promax)/liter drinking water for 4 hours in everyday morning. No additives were added to drinking water in control group. Fresh and clean water was made available at all times. The feeder and drinker were placed in such a way that the broilers were able to eat and drink conveniently. Refusals of the feed were measured weekly in the morning. Feeders were cleaned in every week and drinkers were cleaned daily prior to water supply. In all cases, ad-libitum feeds were supplied to the broilers.

3.7.2 Litter management

Fresh rice husk was used as litter material and extend over the floor at a depth of about 5-6 cm. After first 2 weeks, upper a part of the litter with droppings was removed and replaced with new litter. After 2 weeks, litter was mixed up in every alternative day to allow it to dry fast and remove harmful gases.

3.7.3 Brooding of chicks

The chicks were brooded in four respective enclosures, each with one 100-watt electric lamp. The chicks were supplied a temperature of 35°C during their first day, which gradually decreased every three days interval until they were one weeks old. An automated thermo-hygrometer was used to measure the temperature and relative humidity within the room.

3.7.4 Lighting management

The birds were exposed to a continuous lighting period of 23 hours,followed by 1 hour dark phase in the first 4 days of brooding. The dark period was increased as the age of broiler increased. After 1st week, dark period was provided 6 hours per day until 4th week. After 4th week dark period again decreased ¾ hours until the end of rearing (5th week).

3.7.5 Vaccination

All the experimental birds were vaccinated against New Castle disease, Infectious Bronchitis and Infectious Bursal Disease at the age of day 4th and 11th respectively. All the vaccines were administered as per manufacturer recommendation.

During the trial period, the following vaccination schedule was followed.

Table 3.5 Vaccination schedule

SL. No.	Age of bird (days)	Name of disease	VaccineName	Doses	Methods of vaccination
1	4	IB+ND	MA5+Clone30	1 eye drop	Eye drops
2	11	IBD	D78	1 eye drop	Eye drops
3	19	IBD (Booster)	D78	1 eye drop	Eye drops

IB- Infectious Bronchitis; ND- Newcastle Disease; IBD- Infectious Bursal Disease

3.8 Biosecurity

All proper bio-security measures both inside and outdoor the research shed were strictly maintained during the experimental period. The experimental shed's access was strictly controlled. At the entrance of research shed, foot bath was maintained, where $TH4^+$ solution was used as disinfectant. A separate footwear and apron were used in the experimental shed to prevent contamination. Necessary fencing was installed around the experimental shed and other redundant cares were taken to stay the birds free from rodents and wild birds, small reptiles or any other animals.

3.9 Processing of broilers

Processing of bird is additionally performed at HSTU poultry farm. At the end of experiment, broilers having close to pen average body weight were selected from each pen for recording meat yield parameters. Birds wereslaughtered and allowed to bleed for 2 minutes and submerged in hot water (51- 55°C) for 120 seconds to lose their feathers, a method known as semi-scalding. The feathers were removed manually (by hand) and the birds were again individually weighed. The head, shank, viscera, giblet (heart, liver, and gizzard), and abdominal fat were then removed for determination of meat yield characteristics. Dressed broilers were cut into different pieces, including the breast, thigh, drumstick, and wing. Finally, for each replication, every cut-up parts were weighed and recorded separately.

3.10 Data collection and record keeping

Following records and calculated data were kept throughout the experimental period.

3.10.1 Body weight

Birds were weighed on the first day of experiment and weekly basis for all birds from each replication. In each replication, the average body weight gain of the

broiler was calculated by subtracting initial body weight from the final body weight.

3.10.2 Body weight gain

The body weight gain of each replication was calculated by subtracting the initial body weight from the final body weight of the birds at weekly basis.

BWG = Final weight – Initial weight

3.10.3 Feed intake

The amount of feed consumed by the birds in a very particular replication of each treatment groups was calculated for each week by subtracting the amount of left over feed from the amount supplied for that particular week.

3.10.4 Feed conversion ratio (FCR)

The feed conversion ratio was computed by dividing the amount of feed ingested bythe amount of body weight gain.

$$FCR = \frac{\text{Feed intake (g)}}{\text{Body weight gain (g)}}$$

3.10.5 Temperature and humidity

Throughout the experiment, temperature and humidity were measured four times each day (8 AM, 12 PM, 4 PM, and 8 PM) with an automated thermo-hygrometer.

3.10.5 Records of dressing yield

During dressing the following parameters were recorded-

1. Live weight
2. Dressed weight
3. Thigh weight
4. Breast meat weight

5. Drumstick weight
6. Wing meat weight
7. Heart weight
8. Liver weight
9. Gizzard weight
10. Head weight
11. Neck weight
12. Abdominal fat weight

3.11 Blood collection

A series of sterile test tubes containing anticoagulant EDTA at a ratio of 1: 10 was taken. Sequential killing was done and blood was collected from 2 birds from each replication through slaughtering. The hematological analyses were performed within two hours of blood collection.

3.12 Collection and preparation of serum samples

About 3-4 ml of blood from each replicate bird was collected in the sterile glass test tubes. Tubes were placed in a slanting position (45° angles) at room temperature for clotting. After 2 hours separated blood serum was transferred to an eppendorf tube and centrifuged at 3000 rpm for 10 minutes. The serum was then transferred into another eppendorf tube and preserved at -20°C until analysis. The eppendorf tubes were marked properly with permanent marker for easy identification during chemical analysis.

3.13 Serum biochemical parameters

Serum lipid profile including cholesterol, triglyceride, high density lipoprotein and low density lipoprotein were measured according to their respective protocols. The details of each is given below-

3.13.1 Determination of serum total cholesterol

Principle:

Cholesterol and its ester are released from lipoproteins by detergents. Cholesterol esterase hydrolysis the esters and H_2O_2 is formed in the subsequent enzymatic oxidation of cholesterol by cholesterol-oxidase. In the last reaction a red dye quinonimine dye is formed of which the intensity is proportional to the cholesterol concentration.

Reactions:

$$\text{Cholesterol esters} + H_2O \xrightarrow{\text{CHE}} \text{Cholesterol} + \text{fatty acids}$$

$$\text{Cholesterol} + O_2 \xrightarrow{\text{CHOD}} \text{4-cholestenon} + H_2O_2$$

$$2H_2O_2 + \text{Phenol} + \text{4-Aminophenazone} \xrightarrow{\text{POD}} + 4H_2O$$

The intensity of the color formed is proportional to the cholesterol concentration in the sample.

Composition of the used reagents	
Pips pH 6.9	90 mmol/l
Phenol	26 mmol/l
Cholesterol esterase (CHE)	1000 U/l
Cholesterol oxidase (CHOD)	300 U/l
Peroxides (POD)	650U/l
4-Aminoantipyrine (4-AP)	0.4 mmol/l
Standard Cholesterol aqueous	200 mg/dl

Procedure:

Total cholesterol was determined using the procedure described by Reactivous GPL; 10 µl ready serum samples were taken in each cuvette (1 cm light path) with the help of micropipette. Then 1000 µl reagent was taken to each cuvette and mixed thoroughly by shaking. The cuvettes were incubated at 37°C for 5 minutes. After incubation, each mixture was placed in the spectrophotometer

(Spectronic, Genesis5, and USA) against the blank reagent at 505 nm wave lengths. Then the results were recorded from display. The result was expressed in mg/dl.

Calculation

Cholesterol Conc. (mg/dl) = (Absorbance sample/Absorbance Standard) x 200

3.13.2 Determination of triglycerides

Principle:

Sample triglycerides incubated with lipoproteinlipase (LPL), liberate glycerol and free fatty acids. Glycerol is converted to glycerol-3-phosphate (G3P) and adenosine-5-diphosphate (ADP) by glycerol kinase and ATP. Glycerol-3-phosphate (G3P) is then converted by glycerol phosphate oxehydrogenase (GPO) to dihydroxyacetone phosphate (DAP) and hydrogen peroxide (H_2O_2). On the last reaction, hydrogen peroxide (H_2O_2) reacts with 4-aminophenazone (4-AP) and p-chlorophenol in presence of peroxidase (POD) to give a red color dye.

Reactions:

Triglycerides + H_2O $\longrightarrow$ Glycerol + Free fatty acids

Glycerol +ATP $\longrightarrow$ G3P +ATP+G3P O_2 DAP +H_2O

H_2O_2 + 4-AP + p-Chlorophenol $\longrightarrow$ Quinone +H_2O POD

The intensity of the color formed is proportional to the triglycerides concentration in the sample

**Composition of the used reagents**	
pH 6.	350 mmol/l
p-Chlorophenol	2 mmol/l
Lipoprotein lipase (LPL)	150000 U/l
Glycerol kinase (GK)	500 U/l
Glycerol-3-oxidase (GPO)	3500 U/l
Peroxides (POD)	440 U/l
4-Aminophenazone (4-AP)	0.1 mmol/l
ATP	0.1 mmol/l

Procedure:

The triglyceride of blood serum is determined by spectrophotometer (Spectronic, Genesis 5, USA) according to the technique described by Trinder (1969).

Calculation:

Triglycerides (mg/dl) = (Absorbance sample/Absorbance STD) x Conc. STD

3.13.3 Determination of HDL

Principle:

The very low density lipoprotein (VLDL) and low density lipoprotein (LDL) from serum are precipitated by phosphotungstate in the presence of magnesium ions. After centrifugation, the supernatant contains high density lipoproteins (HDL). The HDL cholesterol fraction is determined using the total cholesterol enzymatic reagent.

**Composition of the used reagents**	
Phosphotungstic acid	14 mmol/l
Magnesium Chloride	2 mmol/l
Standard	50 mg/dl

Procedure:

200 µl serum samples were mixed with 500 µl reagents in the test tube. Then the mixture was allowed uninterrupted for 10 minutes at room temperature. After incubation, each mixture was placed in the spectrophotometer (Spectronic, Genesis 5, USA) against the blank reagent at 505 nm wave lengths.

Calculation:

HDL (mg/dl) = (A 505 nm) Sample read×320

3.13.4 Determination of LDL

The LDL was determined by subtracting the HDL value from the subtracted value of triglyceride from total serum cholesterol that was divided by five.
LDL (mg/dl) = (Total serum cholesterol-Triglyceride/5) x HDL

3.14 Economic appraisal:

The cost-benefit tool was used to calculate the total costs, unit cost of production, FCR, meat-feed ratio, gross income and net income. Production cost was calculated by considering the expenses of chicks, feed, probiotic, feeder, drinker, litter, other equipment, vaccine etc. Feed, chick, litter and vaccination cost were considered as the expenses involved during the experiment.

3.15 Statistical analysis

All recorded and calculated data of the experiment were statistically analyzed using Analysis of Variance (ANOVA) technique in accordance with the principles of Completely Randomized Design (CRD). Duncan Multiple Range Test procedure was used to determine the significant differences among different means at 5% significance level (SAS, 2002).

CHAPTER 4

RESULTS

The experiment was carried out to find out the effect of a commercial citric acid, synbiotic and probiotic treated drinking water on the growth performance, biochemical parameters and cost-benefit analysis of Cobb 500 broiler chicks. The results obtained from the study are showed below in this chapter.

4.1 Body weight and body weight gain

Body weight and body weight gain of broilers fed different diets are presented in **Table 4.1.**

Table 4.1: Growth performance of broiler in different dietary treatments at different ages (DOC-35 days)

Parameter	Treatments				P-value
	Control	Citric acid	Synbiotic	Probiotic	
**Body weight (g/bird)**					
**Initial wt**	45.7±0.03	44.3±0.08	44.6±0.02	45.5±0.05	0.923
**1st week**	206.78±2.15	211.53±1.92	209.58±2.83	212.57±1.16	0.294
**2nd week**	541.15±6.09	548.67±6.98	548.67±4.98	551.23±2.68	0.607
**3rd week**	1062.46±4.41	1051.47±17.24	1047.32±3.73	1069.68±4.80	0.377
**4th week**	1482.76^{c}±1.63	1496.65^{bc}±5.15	1503.86^{b}±8.48	1533.52^{a}±5.66	0.002
**5th week**	1741.17^{b}±0.98	1771^{a}±11.51	1772.68^{a}±10.90	1792.16^{a}±5.89	0.018
**Body weight gain (g/bird)**					
**1st week**	161.06±2.56	167.23±4.38	164.98±2.78	167.07±3.69	0.726
**2nd week**	334.37±4.05	337.14±5.06	339.09±5.22	338.66±2.43	0.864
**3rd week**	521.31±8.97	502.80±22.76	498.65±1.29	518.45±7.46	0.542
**4th week**	420.30^{b}±3.18	445.18^{ab}±12.18	456.54^{a}±4.76	463.84^{b}±8.24	0.021
**5th week**	258.41^{a}±2.44	274.59^{ab}±6.52	268^{b}±2.53	258.64^{b}±3.39	0.047
**Final wt**	1695.47±8.66	1726.70±5.23	1728.08±10.78	1746.66±7.64	0.027

a, b and c values with different superscripts in the same row differ significantly (P<0.05). Data are presented as mean ± SE.

Result from Table 4.1 indicates that there was no significant difference in initial to 3rd week body weight among the dietary groups, but the body weight in 4th and 5th week showed significant differences ($P<0.05$). At the 4th week probiotic group showed the highest body weight (1533.52 g) compare to the control (1482.76 g) group. At the end of the experiment, the highest body weight was found in probiotic group (1792.16 g), followed by synbiotic group (1772.68 g), citric acid group (1771.0 g) and control group (1741.17 g).

There was a significant variation ($P<0.05$) on body weight gain at 4th, 5th week and final period among the different dietary groups. At 4th week of age probiotic group (463.84 g) showed higher body weight gain compared to the control group. Citric acid group (274.59 g) showed higher body weight gain at 5th week of age compared to the control (258.41 g) group. At the end of the experiment, the highest body weight gain was found in probiotic group (1746.66 g) compare to other dietary groups.

However, there was no significant effect in the 1st week; 2nd week and 3rd week body weight gain among the different groups.

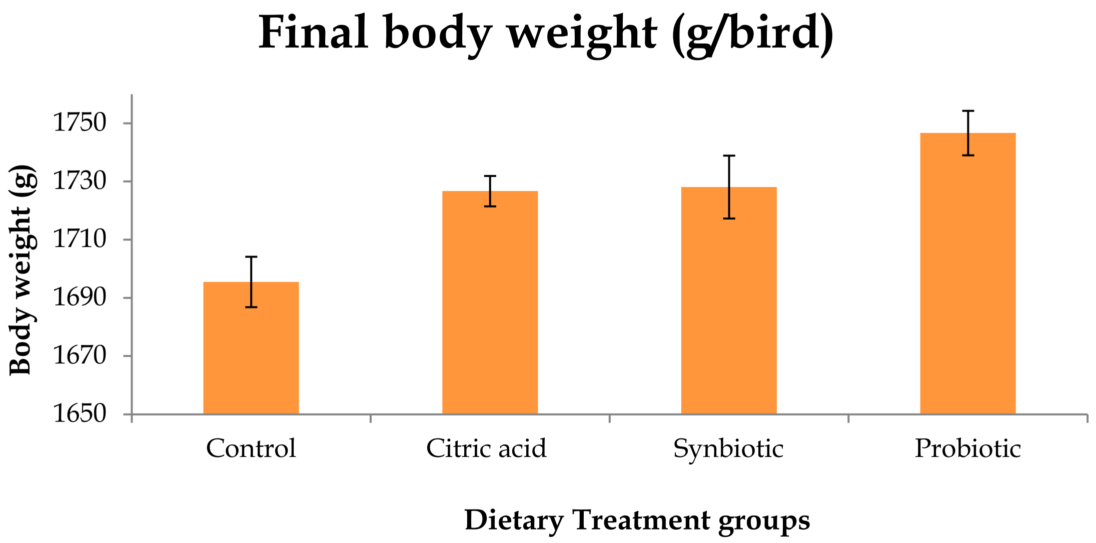

Figure 4.1: Final body weight of broiler in different treatments at different ages

4.2 Feed intake

Data revealed in Table 4.2 that there was no significant effect on 1st, 2nd, 3rd and 4th week feed intake over the other dietary groups. But feed intake in 5th week and overall feed intake showed significant variation (P<0.05) among dietary groups. At the end of the experiment highest feed intake was found in control (2984.18 g) group, followed by citric acid (2934.44 g), symbiotic (2920.2 g) and probiotic (2904.75) group.

Table 4.2: Feed intake (g/bird) of broiler in different dietary treatments at different ages (DOC-35 days)

Parameter	Treatments				P-value
	Control	Citric acid	Synbiotic	Probiotic	
1st week	167.50±1.33	170.57±4.51	166.65±3.47	162.06±5.25	0.856
2nd week	537.34±4.71	535.11±4.29	535.94±2.94	534.12±1.77	0.932
3rd week	767.93±2.45	770.44±0.66	770.14±0.93	771.50±0.36	0.366
4th week	829.70±1.29	826.52±1.53	828.35±0.41	827.78±0.61	0.279
5th week	681.71^{a}±3.41	631.80^{b}±4.74	619.13^{bc}±4.59	609.29^{c}±6.62	0.00
Total	2984.18^{a}±12.4	2934.44^{b}±10.09	2920.2^{b}±8.78	2904.75^{bc}±14.56	0.03

a, b and c values with different superscripts in the same row differ significantly (P<0.05). Data are presented as mean ± SE.

4.3 Feed conversion ratio

The FCR of broiler chickens of different treatment groups recorded during different stages of growth are presented in Table 4.4. Data revealed that there was significant variations (P<0.05) on total feed conversion ratio among different dietary groups.

At the end of the experiment, significantly better FCR (P<0.05) was in probiotic group (1.62) compared to control (1.74). However, citric acid group (1.66) and symbiotic group (1.65) showed no significant difference. The Table also shows

that there was no significant variation in 1st, 2nd and 3rd weeks among different treatment groups. But at 4th and 5th weeks of age probiotic group showed significantly ($P<0.05$) better FCR compared to control group. In overall period, better FCR was found in probiotic compared to other treated groups.

Table 4.3: Feed conversion ratio of broiler in different dietary treatments at different ages (DOC-35 days)

Parameter	Treatments				P-value
	Control	Citric acid	Synbiotic	Probiotic	
1st week	1.04±0.02	1.02±0.02	1.01±0.01	0.97±0.02	0.873
2nd week	1.61±0.00	1.59±0.01	1.58±0.02	1.58±0.02	0.481
3rd week	1.47±0.02	1.54±0.07	1.54±0.00	1.49±0.04	0.506
4th week	1.97^{a}±0.01	1.85^{b}±0.05	1.81^{b}±0.02	1.78^{a}±0.06	0.012
5th week	2.64^{a}±0.02	2.3^{b}±0.06	2.3^{b}±0.03	2.25^{a}±0.02	0.00
Total	1.74^{a}±0.03	1.66^{b}±0.08	1.65^{b}±0.04	1.62^{b}±0.09	0.042

a and b values with different superscripts in the same row differ significantly ($P<0.05$). Data are presented as mean ± SE.

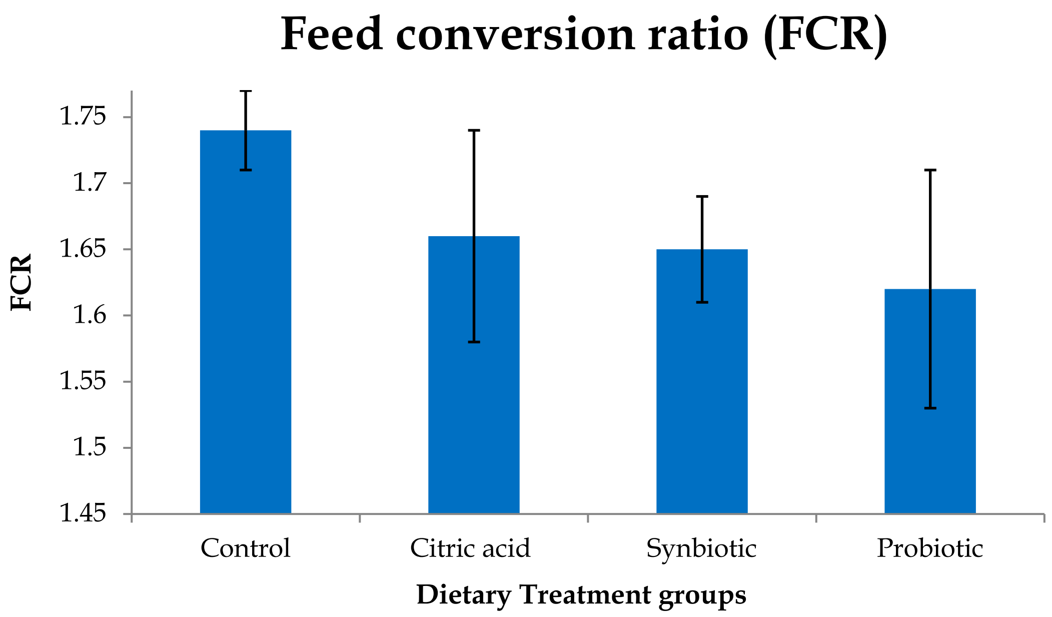

Figure 4.2: Feed conversion ratio of broiler in different treatments at different ages

4.4 Meat yield and bone development

Table 4.4 indicates that there was no significant difference in meat yield and bone development of broilers among dietary treatment groups. Citric acid, synbiotic and probiotic groups showed numerically higher dressing percentage compared to control.

Table 4.4: Meat yield and bone development of broiler in different dietary treatments

Parameter	Treatments				P-value
	Control	Citric acid	Synbiotic	Probiotic	
**Meat yield (% in relation to body weight)**					
DP (%)	58.37±2.24	57.10±1.32	56.82±0.78	58.41±0.47	0.780
Breast meat	20.12±0.84	17.89± 0.35	18.36±0.68	20.15±0.80	0.103
Thigh meat	8.12±0.13	7.77±0.12	7.51±0.14	8.07±0.51	0.117
Drumstick meat	6.39±0.24	5.54±0.19	5.30±0.08	6.02±0.40	0.062
Wing meat	3.44±0.04	3.27±0.05	3.32±0.05	3.39±0.03	0.058
**Bone development(% in relation to body weight)**					
Thigh bone	2.01±0.15	2.04±0.05	1.97±0.04	1.93±0.18	0.911
Drumstick bone	2.84±0.06	2.72±0.07	2.88±0.17	2.84±0.04	0.691
Wing bone	2.84±0.06	2.91±0.00	2.81±0.09	3.07±0.29	0.066

Data are presented as mean ± SE.

4.5 Dressing parameters of broiler

Result from Table 4.5 indicated no significant differences in head, neck, heart, and leg weight in relation to body weight among different dietary groups.

Table 4.5: Dressing parameters of broiler in different dietary treatments (% in relation to body weight).

Parameter (%)	Treatments				P-value
	Control	Citric acid	Synbiotic	Probiotic	
Head weight	1.31±0.04	1.35±0.01	1.410±0.05	1.52±0.01	0.077
Neck weight	2.25±0.14	2.04±0.02	1.99±0.03	1.93±0.24	0.463
Leg weight	2.30±0.05	2.51±0.04	2.39±0.09	2.65±0.07	0.062
Liver weight	2.49±0.02	2.39±0.19	2.32±0.10	1.99±0.05	0.059
Heart weight	0.65±0.09	0.62±0.02	0.50±0.03	0.51±0.03	0.136
Gizzard weight	1.60^{b}±0.02	1.70^{a}±0.04	1.49^{c}±0.01	1.71^{a}±0.02	0.001
Abdominal fat	1.28^{a}±0.08	0.89^{c}±0.04	1.13^{b}±0.07	0.78^{c}±0.04	0.039

a, b and c values with different superscripts in the same row differ significantly (P<0.05).
Data are presented as mean ± SE.

Probiotic group showed the higher head (1.52%), leg (2.65%), gizzard (1.71%) and the lower neck (1.93%), liver (1.99%), abdominal fat (0.78%) weight compare to control group. Control group showed significantly higher abdominal fat weight (1.28%) as compared to different dietary treatments. However, probiotic group showed significantly (P<0.05) highest gizzard weight compared to control and other treated groups.

4.6 Serum biochemical parameters

Table 4.6 indicates that there were no significant difference in TG and HDL among different treatment groups. Probiotic groups showed significantly

highest (P<0.0.05) cholesterol and LDL compared to control and other treated groups. Citric acid group showed numerically lowest cholesterol and LDL compared to control and synbiotic group. Numerically lowest HDL (33.48 mg/dl) and highest TG (82.03 mg/dl) level found in probiotic group, whereas lowest TG level found in control group.

Table 4.6: Blood biochemical parameters of broiler in different dietary treatments (mg/dl)

Parameter	Treatments				P-value
	Control	Citric acid	Synbiotic	Probiotic	
**Total Cholesterol**	112.62^{b}±5.94	101.29^{b}±18.61	108.96^{b}±1.53	138.83^{a}±11.89	0.013
**TG**	65.30±2.35	67.34±4.00	77.95±5.89	82.03±5.42	0.090
**HDL**	37.06±1.10	37.76±2.06	37.28±0.14	33.48±0.14	0.107
**LDL**	62.62^{b}±6.58	50.06^{b}±17.36	56.08^{b}±0.22	88.93^{a}±10.94	0.039

a and b values with different superscripts in the same row differ significantly (P<0.05). Data are presented as mean ± SE.

4.7 Economic appraisal

The cost involved in rearing broilers up to 28 days and the income obtained by selling broilers along with four different treatment groups were calculated and present in the Table 4.7.

Table 4.7 Cost benefit ratio of broiler in different dietary treatments

Parameter	Treatments			
	Control	Citric acid	Synbiotic	Probiotic
a. Feed intake (g/broiler)	2984.18	2934.44	2920.2	2904.75
b. Final body wt. (g/broiler)	1741.17	1771	1772.68	1792.16
c. Feed price (tk/kg)	65	65	65	65
d. Cost for citric acid	0	0.9	0	0
e. Cost for synbiotic	0	0	0.82	0
f. Cost for probiotic	0	0	0	0.7
g. Total feed cost (c+d+e+f)/kg	65	65.9	65.82	65.7
h. Feed cost/bird	193.97	193.37	192.20	190.84
i. Chick cost tk/bird	9	9	9	9
J. Miscellaneous (vaccines, disinfectant, transport, bedding materials, labor)	20	20	20	20
k. Total cost of production/live bird	222.97	220.37	221.20	219.84
l. Total cost of production tk./kg live bird	128.05	124.43	124.78	122.66
m. Scale value/kg	155.01	155	155.05	155.01
Benefit cost ratio (BCR)	1.21	1.24	1.24	1.26

The total cost of production per kg feed in different treated group is slightly higher as compared to the control. The cost of production per kg broiler was slightly lower in additives group than control group. Moreover, when the live broiler was sold at tk. 155 per kg, then the income per kg live bird was higher in all supplemental groups compared to the control group.

The profit both in terms of per broiler and per kg broiler was higher in probiotic followed by synbiotic, citric acid and control group respectively. It is therefore clear that the supplementation of probiotic through drinking water is more profitable than other treatments.

CHAPTER 5

DISCUSSION

5.1 Body weight and body weight gain

It is obvious from the data that antibiotic alternative additives such as citric acid, probiotic, and synbiotic in broiler water significantly improved body weight and weight gain when compared to the control group throughout the experimental period. Among the additives supplemented groups highest body weight was found in probiotic group. Probiotic is a type of microbial feed additives. The main benefit of using probiotic via drinking water is that microorganisms are not destroyed by probiotic water. Once after applied in the broilers, the feed additives start to act upon their digestibility of the ingested feed materials, which are then helped the birds to utilize feed more efficiently. It can be assumed that, the efficient utilization of feed by the broilers might give rise to better growth performance of the broiler chickens.

In case of probiotic, these results are in the agreement with the findings of Eckert *et al.,* (2010), demonstrated that application of a probiotic (Protexin) via drinking water increased body weight on day 15 and day 40 compared to all other treatments. Jayakumar *et al., (*1996) reported that the increase in weight and better quality of broiler meat with the use of probiotics in poultry rations. While several research have claimed a significant improvement in body weight of broilers following probiotic supplementation in broiler diet (Gohain and Sapcota, 1998), there are others who have terminated supported their studies that addition of probiotics didn't significantly affect the weight of broilers (Samanta and Biswas, 1995). The beneficial effect of probiotic supplementation to broiler in terms of increased body weight and body weight gain is well documented by Singh *et al.,* (1999). Jin *et al.,* (1998) observed that dietary inclusion of probiotics Lactobacillus has been reported to enhance body weights and feed to gain ratio when compared to the control broilers that is similar to our result. In addition, probiotics supplementation to

diet improved feed intake, feed efficiency, and carcass yield of broilers (Denil *et al.,* 2003). Improvements in growth performance are frequently attributed to the composition and activity of the gut microflora which regulate nutrient utilization (Yang *et al.,* 2009). Kalavanthy *et al.,* (2003) found that administering probiotics in drinking water is reported to result in a smaller increase in average daily gain compared with administering them via feed.

Synbiotic group showed significantly better growth rate compared to the control group. These results are in the agreement with the findings of Al-Sultan *et al.* (2016) who found that synbiotics significantly increased the bird's weight and weight gain as compared to the control group. Suparom *et al.,* (2013) found that supplementing synbiotics in broiler diet improved production performance but had no influence on carcass quality. Morever, Awad *et al.*, (2008) reported that feeding broilers with synbiotics (Biomin®IMBO comprising *Enterococcus faecium* and oligosaccharide) enhanced average body weight gain and feed conversion ratio.

Present studies showed that the inclusion of citric acid enhanced growth rate of broiler. These results are in concordance with the findings by Moghadam *et al.,* (2006) who observed that better live weight of broiler due to citric acid supplementation. Abdel-Fattah *et al.,* (2008) reported that significantly ($p<0.05$) improved live body weight, weight gain of broilers with supplemental citric acid, acetic acid, and lactic acid compared to control. This may be due to decreasing the pH in gastrointestinal tract with citric acid and growth inhibition of potential pathogenic bacteria eg. *E coli,* and Salmonella in the feed and in gastrointestinal tract. Beneficial effect of citric acid on weight gain of broilers was also reported by Nezhad *et al.,* (2007), Shen *et al.,* (2005), Andrys *et al.,* (2003), and Ivanov (2005). Chowdhury *et al.*, (2009) found highest live weight and weight gain in citric acid group than all other groups of broiler. Kopecky *et al.*, (2012) reported that broiler drinking water supplementation with citric acid can increase the body weight gain, improve feed efficacy and immune response. On the contrary, Mohyla *et al,*. (2007) reported that inclusion of CA from 0% to

0.4% in drinking water had no effect on growth performance of broilers from 35 to 42 day of age.

5.2 Feed intake

Results of the present study demonstrated that probiotic group numerically intake more feed than control group until 3rd week of age. These results are in the agreement with the findings of Edens (2003) who stated that probiotic dietary group significantly increases feed intake of broiler. Mohan *et. al.* (1996) also indicated that probiotic supplemented diets improved the feed intake irrespective of seasons. But several researchers (Panda *et al.*, 2008; Faria *et al.*, 2009) found no significant difference in feed intake between control and probiotic group.

Synbiotic group presented significantly lower feed intake as compared to control group whereas Abdel-Raheem *et al.,* (2012); Shokrii *et al.,* (2017); Min*et al,.* (2016) found that synbiotic supplementation in broiler diets significantly ($P<0.05$) increased the feed intake as compared to the control group.

Citric acid group showed significantly higher feed intake than probiotic group. It has been reported that dietary citric acid increases feed consumption (Moghadam *et al.,* 2006; Atapattu and Nelligaswatta, 2005, Philipsen 2006) reported that supplementation of organic acids such as citric acid in drinking water helps to reduce the level of pathogens in water, crops and the proventriculus, regulate the gut microflora, increase feed digestion and improve growth performance of birds.

5.3 Feed conversion ratio

In this study, dietary supplementation of probiotic group had better FCR as compared to the control and other additives group. The present results of study are in agreement with the findings of Dhande *et al.,* (1993) and Verma (1992), who found significant differences in FCR of birds fed probiotics. Timmerman *et al.,* (2006) also found that the administration of probiotics via the drinking water

had beneficial effects on broiler performance and probiotic treatment significantly improved feed conversion ratio. Anjum *et al.,* (2005) investigated the effects of a multi-strain probiotics (protexin) on broiler growth performance and showed that FCR significantly improved FCR in chicks fed on protexin supplemented diets compared to control diets.

Result of present study showed that synbiotic group has significantly better FCR compared to control group. This is in concordance with studies by Raksasiri *et al.,* (2018); Suparom *et al.,* (2013); Mountzouris *et al*., (2007); Awad *et al.,* (2008). They found better FCR of broilers with treated symbiotic.

Citric acid group showed significantly better FCR compared to control group. This finding is in agreement with several researchers namely, Chowdhury *et al.,* (2009), Abdel- Fattah *et al.,* (2008) and Nezhad *et al,.* (2007). They observed profound effect of citric acid on feed conversion in broilers. FCR was significantly improved in broiler chickens with flavomycin when compared to control group (Ashayerizadeh *et al*., 2009). Moreover, Hassan *et al.,* (2010) stated that organic acids significantly ($p<0.01$) improved feed conversion ratio.

5.4 Meat yield, bone development and different dressing parameters

Results of the present study showed that there is no significant effect of synbiotic, probiotic, and citric acid supplementation in broiler dressing percentage, meat yield (breast meat, thigh meat and wing meat) and bone development (thigh bone, drumstick bone, wing bone) compared to control but significant variation was found liver, gizzard, abdominal fat in relation to body weight among different dietary groups. Dressing percentage, breast, wing meat and bone development percentage were high in probiotic treated groups compared to control group. This results are in agreement with the reports of previous researchers (Allahdo *et al*., 2018), who found that broilers diet supplemented with probiotic showed increased meat yield and bone development than the control. The difference between two studies is to

variation in sources and application of probiotics. In our study, we supplied probiotics through drinking water, whereas they applied probiotics with diet. Hossain *et al.,* (2012) reported that the addition of probiotics increased breast meat absolute and relative weight. Moreover, Warsito *et al.*, (2019) demonstrated that the Combination of citric acid-dextrose in drinking water could increase the percent yield of broiler carcasses and immune response. Control group showed higher carcass weights (Neck, liver, heart, abdominal fat) percentage than probiotic groups. But Anjum *et al.*, (2005) did not find any difference carcass percentage between a control and a probiotic group. Sarangi *et al.,* (2016) showed that the supplementation of synbiotics did not affect the dressing percentage.

Gizzard weight is increased when birds are fed with citric acid supplement compared to control group. The result of this study is accordance with Aksu *et al.,* (2007) has noted that internal edible organ weights can be improved by organic acid supplementation at 4 g/kg of feed. Izat *et al.,* (1990) who reported that addition of buffered propionic acid @ 0.4% in the diet of broilers had no effect on organ weight.

In this study there is no significant difference among different treatment for the reduction of abdominal fat. This result is generally contradictory with those of Panda *et al.,* (2009), who reported that abdominal fat content can be significantly reduced by butyric acid treatment compared to control or antibiotic treatment, and Lessard *et al.* (1993), who observed that pyruvic acid or citric acid supplementation reduces abdominal fat content.

5.5 Serum biochemical parameters

A significant difference was observed in blood cholesterol and LDL when used different additives in different treatment groups in the drinking water of broilers. Probiotic group showed significantly highest cholesterol and LDL compared to control and other treated groups. This result is contradictory to the

studies by *Torshizi et al.,* (2010) who reported that probiotic supplied via drinking water reduced plasma cholesterol and triglyceride concentrations compared to the control and other treated groups. Panda *et al.,* (2006) also reported that dietary supplementation of probiotic *Lactobacillus sporogenes* lowered serum level of total cholesterol, low-density lipoprotein cholesterol, very low-density lipoprotein cholesterol and triglycerides. The increased amount of total cholesterol and LDL in probiotic supplemented group in the present result is confusing and not able to explain clearly about the mode of action of probiotic on cholesterol.

In the result, symbiotic group showed higher HDL in symbiotic group compared to control group which coincides with the findings of Tufan *et al.,* (2017) reported that adding of synbiotics in feed improve the blood HDL level. Citric acid group showed significantly lowest cholesterol and LDL level compared to control and other treated groups. Oppositeresults were found by EL-Afifi *et al.,* (2001) as they observed no significant effect on cholesterol and LDL in the broiler chicken fed on citric acid.

Although there are a few findings on the changes in serum biochemical parameters due to the application of different feed additives with feed, but there are no such research on the changes when the additives are fed to broilers with drinking water. As a result, feeding different additives via drinking water didn't provide clear evidence about the changes in serum biochemical properties of broiler.

5.6 Cost-effectiveness of production

The cost of production was calculated considering the cost of chicks, feed, additives and medicine. It is evident that the cost of production of broiler was lower in probiotic group than control. Profit is the main goal of every business, and it may be fixed by lowering inputor raising output. Economic data clearly indicated that probiotic supplementation via drinking water was more profitable and economical to attain maximum gains from broiler

production. This result is similar to findings of Roy *et al.,* (2013) who reported that feeding probiotic to broiler was either similar or more profitable than combination of probiotic + AGP while better than AGP alone. Better efficiency of feed utilization with probiotics in broiler drinking water was a major factor which resulted in decreased cost of production. The results of the present study are in agreement with the findings of Anjum *et al.,* (2005), who reported that feed cost per kilogram broiler produced was less in probiotic supplemented groups compared to non-supplemented group.

`

CHAPTER 6

SUMMARY AND CONCLUSION

An experiment was carried out with 400 day old straight-run Cobb 500 broiler chicks for a period of 35 days of age at Hajee Mohammad Danesh Science and Technology University (HSTU) Poultry Farm, Dianjpur to evaluate the beneficial effect of different water additives on growth performance, blood biochemical parameters and meat yield characteristics of broilers. The chicks were divided into 4 dietary groups and each group was replicated to 4 sub-groups each of 25 birds. The dietary groups were control (without additives), citric acid, synbiotic and probiotic group. Throughout the experimental period live weight, weight gain, feed intake, feed conversion ratio, meat yield, bone development, blood biochemical parameters of broiler on different treatments were recorded and statistically analyzed.

The growth performance parameters such as bodyweight, weight gain, feed intake and mortality were taken weekly basis. There were no significant differences on body weight and body weight gain among the dietary groups from day old to 3rd week of age. After 3rd week body weight and body weight gain showed significant difference among the dietary groups. Numerically at the end of the experiment highest body weight was found in probiotic group (1792.16 g) followed by symbiotic group (1358.98 g), citric acid group 1771.0 g) and control group (1278.25 g). Considering the total body weight gain probiotic group (1746.66 g) showed the highest body weight gain while the lowest body weight gain found in control (1695.47 g) group.

There was no significant difference on feed intake upto 4 weeks of age. But feed intake on 5th week and overall feed intake showed significant variation among different dietary groups. Citric acid fed groups showed higher feed intake compared to other dietary groups.

The FCR of broilers differed significantly during 4th and 5th week

respectively. At the end of the experiment, better FCR was observed in the probiotic group (1.62) compared to the synbiotic (1.65), citric acid (1.66) and control (1.74) group. There were no significant difference in dressing percentage, meat yield (breast meat, thigh meat, drumstick meat and wing meat) and bone development (thigh, wing and drumstick bone).

In present study, probiotic groups showed significantly highest (P<0.0.05) cholesterol and LDL compared to control and other treated groups. Citric acid group showed numerically lowest cholesterol and LDL compared to control and synbiotic group. Numerically lowest HDL (33.48 mg/dl) and highest TG (82.03 mg/dl) level found in probiotic group, whereas lowest TG (65.30 mg/dl) level found in control group.

The cost of production per kg live bird was lower in probiotic, synbiotic and citric acid groups as compared to control. Consequently probiotic, synbiotic and citric acid groups showed higher profitability compared to control group. Moreover when the live broiler was sold at TK.155 per kg then the income per kg live bird was higher in probiotic group compared to the control group.

However, an overview of the results obtained in this study revealed that supplementation of citric acid, synbiotic and probiotic through drinking water to broiler chickens enhance body weight, body weight gain, better FCR and nutrient availability of birds. The use of antibiotics alternative such as citric acid, synbiotic and probiotic helps to produce more safe and reliable poultry meat than conventionally raised broilers. Nevertheless, probiotic could be employed as the best additive among the other additives which are commonly used in drinking water (Citric acid, synbiotic) for broiler production. It is suggested that supplementation of probiotic through drinking water on broiler chicken might be more economical and it could enhance commercial production.

REFERENCES

Abdel-Fattah SA, EI-Sanhoury MH, EI-Mednay NM, Abdul-Azeem F 2008: Thyroid activity of broiler chicks fed supplemental organic acids. *International Journal of Poultry Science* **7** 215–222.

Abdelqader A, Al-Fataftah AR, Das G 2013: Effects of dietary Bacillus subtilis and inulin supplementation on performance, eggshell quality, intestinal morphology and micro flora composition of laying hens in the late phase of production. *Animal Feed Science Technology* **179** 103-111.

Abdel-Raheem SM, Abd-Allah SM, Hassanein KM 2012: The effects of prebiotic, probiotic and synbiotic supplementation on intestinal microbial ecology and histomorphology of broiler chickens. *International Journal of Agronomy and Veterinary Medicine Science* **6** 277-289.

Adil S, Banday MT, Bhat GA, Qureshi SD, Wani SA 2011: Effect of supplemental organic acids on growth performance and gut microbial population of broiler chicken. *Livestock Research for Rural Development* **23(1)** 1-8.

Afsharmanesh M, Pourreza J 2005: Effect of calcium, citric acid, ascorbic acid, vitamin D3 on the efficacy of microbial phytase in broiler starters fed wheat-based diets on performance, bone mineralization and ileal digestibility. *International Journal of Poultry Science* **4** 418–424.

Ahmad I, 2006: Effect of probiotics on broilers performance. *International Journal of Poultry Science* **5**(6) 593-597

Aksu T, Ates CT, Erdogan Z, Baytok E 2007: The response of broilers to dietary organic acid mixture. *Indian Veterinary Journal* **84** 385-387.

Alkhalf A, Alhaj M, Al-Homidan I 2010: Influence of probiotic supplementation on immune response of broiler chicks. *Egypt Poultry Science* **30** 271-280.

Al-Sultan SI, Abdel-Raheem SM, El-Ghareeb WR, Mohamed MH 2016: Comparative effects of using prebiotic, probiotic, synbiotic and acidifier on growth performance, intestinal microbiology and

histomorphology of broiler chicks. *Japanese Journal of Veterinary Research* **64** 187-195

Amerah AM, Quiles A, Medel P, Sanchez J, Lehtinen MI 2013: Effect of pelleting temperature and probiotic supplementation on growth performance and immune function of broilers fed maize/soy-based diets. *Animal Feed Science and Technology* **180** 55-63.

Anjum MI, Khan AG, Azim A, Afzal M 2005: Effect of dietary supplementation of multi strain probiotic on broiler growth performance. *Pakistan Veterinary Journal* **25**(1) 20-25.

Al-Sultan SI, Abdel-Raheem SM, El-Ghareeb WR, Mohamed MH 2016: Comparative effects of using prebiotic, probiotic, synbiotic and acidifier on growth performance, intestinal microbiology and histomorphology of broiler chicks. *Japanese Journal of Veterinary Research* **64** 187-195.

Allahdo P, Ghodraty, J 2018: Effect of probiotic and vinegar on growth performance, meat yields, immune responses, and small intestine morphology of broiler chickens. *Italian Journal of Animal Science* **17** 675-685.

Andrys R, Klecker D, Zeman I and Marecek E 2003: The effects of changed pH values of feed in isophosphonic diets on chicken broiler performance. *Czech Journal of Animal Science* **48** 197-206.

Ao T, Cantor AH, Pescatore AJ, Ford MJ, Pierce JL, Dawson KA 2009 Effect of enzyme supplementation and acidification of diets on nutrient digestibility and growth performance of broiler chicks. *The Journal of Poultry Science* **88(1)** 111-7.

Apleblat A 2014: Properties of citric acid and its solutions 13-141

Ashayerizadeh A, Dabiri N, Mirzadeh KH, Ghorbani MR 2011: Effect of dietary supplementation of probiotic and prebiotic on growth indices and serum biochemical parameters of broiler chickens. *Journal of Cell and Animal Biology* **58** 152-156.

Atapattu NSBM, Nelligaswatt CJ 2005: Effect of citric acid the performance and utilization of phosphorus and crude protein in broiler chicken fed rice by products based diets. *International Journal of Poultry Science* **4** 990-993.

Awad WA, Ghareeb K, Boehm J 2008: Intestinal structure and function of broiler chickens on diets supplemented with a synbiotic containing Enterococcus faecium and oligosaccharides. *International Journal of Molecular Sciences* **9** 2205- 2216.

Bansal GR, Singh VP, Sachan N (2011): Effect of Probiotic Supplementation on the Performance of Broilers. *Asian Journal of Animal Sciences* **5** 277-284.

Boling SDM, Webel IM, Parsons CM and Baker DH 2000: Effect of citric acid on phytate phosphorus utilization in young chicks and pigs, *Indian Journal of Animal Science* **78** 682–689.

Brugaletta G, Cesare AD, Zampiga M, Laghi L, Oliver C, Zhu C, Manfreda G, Syed B, Valenzuela L and Sirri F 2020: Effects of alternative administration programs of a synbiotic supplement on broiler performance, footpad dermatitis, caecal microbiota, and blood metabolites. *Animals* **10** 522.

Brzoska F, Sliwinski B, Michalik-Rutkowska O 2013: Effect of dietary acidifier on growth, mortality, post-slaughter parameters and meat composition of broiler chickens. *Annals of Animal Science* **13** 85–96.

Bywater RJ 2005: Identification and surveillance of antimicrobial resistance dissemination in animal production. *Poultry Science* **84** 644-648.

Cao GT, Zeng XF, Chen AG, Zhou L, Zhang L, Xiao YP, Yang CM 2013: Effects of a probiotic, *Enterococcus faecium* on growth performance, intestinal morphology, immune response, and cecal microflora in broiler chickens challenged with Escherichia coli K88. *Poultry science* **92**(11) 2949-55.

Capcarova M, Kolesarova A, Massanyi P, Kovacik J 2010: Selected blood biochemical and haematological parameters in turkeys after an experimental probiotic *Enterococcus Faecium* M–74 strain administration. *International Journal of Poultry Science* **7** 1194-9.

Casewell M, Christian F, Marco F, McMullin P, chPhillips I 2003: The European ban on growth-promoting antibiotics and emerging consequences for human and animal health. *Journal of Antimicrobial Chemotherapy* **52** 159–161.

Cavazzoni V, Adami A, Castrovilli C 1998: Performance of broiler chickens supplemented with Bacillus coagulans as probiotic. *British Poultry Science.* **39**(4): 526-529.

Celik K, Denil M and Okan F 2003: Effect of dietary probiotic, organic acids and antibiotic supplementation of diets on broiler performance and carcass yield. *Pakistan journal of Nutrition* **2** 89-91.

Chowdhury R, Islam KMS, Khan MJ, Karim MR, Haque MN, Khatun M, Pesti GM 2009: Effect of citric acid, avilamycin, and their combination on the performance, tibia ash, and immune status of broilers. *Poultry Science* **88** 1616–1622.

Collins MD, Gibson GR 1999: Probiotics, prebiotics, and synbiotics: approaches for modulating the microbial ecology of the gut. *The American Journal of Clinical Nutrition* **69** 1052S-1057S.

Denil M, Okan F, Celik K 2003, Effect of dietary probiotic, organic acids and antibiotic supplementation of diets on broiler performance and carcass yield. *Pakistan Journal of Nutrition* **2** 89-91.

Dhande VU, Kukde RJ, Lende RM and Sarode DB 1993: Effect of giprobiotic on performance of broilers. *Poultry Guide* **30** 39-41.

Eckert NH, Lee D, Hyatt SM, Stevens S, Anderson PN, Anderson R, Beltran G, Schatzmayr M, Mohnl DJ and Caldwell 2010: Influence of probiotic administration by feed or water on growth parameters of broilers reared on medicated and non-medicated diets. *Journal of Applied Poultry Research* **19** 59-67.

Edens FW 2003: An alternative for antibiotic use in poultry probiotics. Brazilian Journalof *Poultry Science* **5** 75–97.

Eidelesburger U, Kirchgessener M 1994: Effect of organic acids and salts in the feed on fattening performance of broilers. *Archiv-Fur- Geflugelkunde* **58** 268-277.

El-Afifi SF, El-Medney NM, Attia M 2001: Effect of citric acid supplementation in broiler diets on performance and intestinal microflora. *Egypt. Poultry Science Journal* **21** 491-505.

Emami NK, Naeini SZ, Ruiz-Feria CA 2013: Growth performance, digestibility, immune response and intestinal morphology of male broilers fed phosphorus deficient diets supplemented with microbial phytase and organic acids. *Livestock Science* **157(2-3)** 506-13.

Ezema C 2013: Probiotics in animal production: A review. *Journal of Veterinary Medicine and Animal Health* **5** 308-316.

Faria DE, Henrique APF and Franzolin R 2009: Alternatives to the use of antibiotic growth promoter for broiler chickens. *Probiotic Ciencia Animal Brasileira* **10** 18-28.

Fascina VB, Sartori JR, Gonzales E, Barros De Carvalho F, Pereira De Souza IMG, Polycarpo GV, Stradiotti AC, Pelícia VC 2012: Phytogenic additives and organic acids in broiler chicken diets. *Revista Brasileira de Zootecnia* **41** 2189–2197.

Fathi M, Al-Homidana I, Al-Dokhaila I, Alsag A 2018: Effects of dietary probiotic (*Bacillus subtilis*) supplementation on productive performance, immune response and egg quality characteristics in laying hens under high ambient temperature. *Italian Journal of Animal Science* **17** 804–814.

Fathi MM, Ebeid TA, Al-Homidan I, Soliman NK, Abou-Emera 2016: Influence of probiotic supplementation on immune response in broilers raised under hot climate. *British poultry science* **58(5)** 512-516.

Falkowski J F, Agerne F X 1984: Fumaric and citric acid as feed additives in statrer pig nutrition. *Journal of Animal Science* **58** 935-938

Fornazier R, Junior VR, Albino LF, Rodrigues D, Tavernari FD, da Silva D, Rostagno H, Serafini S 2019: A Symbiotic Improves Performance and Carcass Yield of Broilers. *Journal of Applied Poultry Research* 28(2) 383-9.

Fuller R 1989: Probiotics in man and animals. *Journal of Biotechnology* **66** 365-378.

Furtula V, Farrell EG, Diarrassouba F, Rempel H, Pritchard J, Diarra MS 2010: Veterinary pharmaceuticals and antibiotic resistance of Escherichia Coli isolates in poultry litter from commercial farms and controlled feeding trials. *Poultry Science* **89** 180–188.

Gaggia F, Mattarelli P, and Biavati B 2010: Probiotics and prebiotics in animal feeding for safe food production. *International Journal of Food Microbiology* **141** S15–S28.

Ghadban GS (2002): Probiotics in broiler production- a review. Archievement fur Geflugelkunde **66(2)** 49-58.

Ghasemi HA, Shivazad M, Esmaeilnia K, Kohram H, Karimi MA 2010: The effects of a synbiotic containing Enterococcus faecium and inulin on growth performance and resistance to coccidiosis in broiler chickens. *The Journal of Poultry Science* **47(2)** 149-55.

Ghazalah AA, Ali AM 2008: Rosemary leaves as a dietary supplement for growth in broiler chickens. *International Journal of Poultry Science* **7** 234-239.

Gohain AK and Sapcota D 1998*:* Effect of probiotic feeding on the performance of broilers. *Indian Journal of Poultry Sci*ence **33** 101-105.

Gong-YiFeng, Liao-Herong, Wang-JinFu and Li-HongYan 2006: Effect of wheat middlings, microbial phytase and citric acid on phytate phosphorus, calcium and protein utilization of broilers. *Agricultural Science in China* **5** 318-322

Gunal M, Yayli G, Kaya O, Karahan N, Sulak O 2006: The effects of antibiotic growth promoter, prebiotic or organic acid supplementation on growth performance, intestinal microflora and tissue of broilers. *International Journal of Poultry Science* **5** 149-155.

Hassan H M A, Mohamed M A, Youssef A W and Hassan E R 2010: Effect of using organic acids to substitute antibiotic growth promoters on performance. *Asian-Australian Journal of Animal Science* **23**(10) 1348–1353.

Hassanpour H, Moghaddam AK, Zamani KM 2013: Effects of synbiotic on the intestinal morphology and humoral immune response in broiler chickens. *Livestock Science* **153** 116–122.

Hill C, Guarner F, Reid G, Gibson GR, Merenstein DJ, Pot B, Morelli L, Canani RB, Flint HJ, Salminen S 2014: Expert consensus document: The International Scientific Association for Probiotics and Prebiotics consensus statement on the scope and appropriate use of the term probiotic. *Nature Reviews Gastroenterology and Herpetology* **11** 506-514.

Irshad A 2006: Effect of probiotics on broilers performance. *International Journal of Poultry Science* **5(6)** 593-7.

Isabel B, Santos Y 2009: Effects of dietary organic acids and essential oils on growth performance and carcass characteristics of broiler chickens. *Journal of Applied Poultry Research* **18** 472.

Islam K 2012: Use of citric acid in broiler diets. *World's Poultry Science Journal* **68** 104-118.

Islam O, Khatun S, Famous M, Uddin M M, Azad S A K, Uddin M N, Khayer A 2019: Productive performance of different broiler strains under the intensive management in Kishoreganj district of Bangladesh. *Mirror of Research in Veterinary Sciences and Animals* **8** (2):13-23

Jayakumar K, Munagowda T, Honnegowda D 1996: Probiotics in poultry nutrition. *Poultry Advisor* **19** 25-26.

Ivanov I 2005: Laboratory study to determine the effect of a probiotic mixture on broilers. *Biotechnology in Animal Husbandry* **21** 107-123.

Izat A L, Colberg M, Thomas R A, Adams M H and Driggers, C D 1990: Effects of lactic acid in processing waters on the incidence of salmonellae on broilers. Journal of Food Quality **13** 295-306.

Jin LZ, Ho YW, Abdullah N, Jalaludin S 1998: Probiotics in poultry: modes of action. *World's Poultry Science Journal* **53** 351-368.

Jozefiak D, Rutkowski A 2005: The effect of supplementing a symbiotic, organic acids or β-glucanase to barley based diets on the performance of broiler chickens. *Journal of Animal and Feed Sciences* **14** 447-450.

Kabir SML 2009: The Role of Probiotics in the Poultry Industry. *International Journal of Molecular Sciences* **10** 3531-3546.

Kalavathy R, Abdullah N, Jalaludin S and Ho Y W 2003: Effects of Lactobacillus cultures on growth performance, abdominal fat deposition, serum lipids and weight of organs of broiler chickens. *British Poultry Science* **44**(1) 139–144.

Kopecky *S* 2012: Effect of ctric acid supplement in drinking water on performance of broiler chickens. *Animal Science Biotechnology* **45** 51-54.

Lessard P, Lefrançois MR, Bernier JF 1993 Dietary addition of cellular metabolic intermediates and carcass fat deposition in broilers. *Poultry Science* **72** 535–545.

Li H, Zhang T, Li C, Zheng S, Li H, Yu J 2020: Development of a microencapsulated synbiotic product and its application in yoghurt **122** 109033.

Lilly DM, Stillwell RH 1965: Probiotics: growth-promoting factors produced by microorganisms. *Poultry Science* **147** 747-8.

Liong MT, Shah NP 2006: Effects of a *Lactobacillus Casei* symbiotic on serum Lipoprotein, intestinal microflora and organic acid in rats. *Journal of Dairy Science* **89** 1390–1399.

Luckstadt C, Steiner T 2007 Effects of phytogenics and organic acids alone and in combination on growth performance of weaned piglets. *Proceedings of the British Society of Animal Science* **2007** 200-200.

Madej JP, Stefaniak T, Bednarczyk M 2015: Effect of in ovo-delivered prebiotics and synbiotics on lymphoid-organs' morphology in chickens. *Poultry Science* **94** 1209–1219.

Maiorka A, Santin AME, Borges SA, Opalinski M, Silva AVF 2004: Evaluation of a mix of fumaric, lactic, citric and ascorbic acids on starter diets of broilers. *Archives of Veterinary Science* **9** 31-37.

Maiorano G, Sobolewska A, Cianciullo D, Walasik K, Elminowska-Wenda G, Sławińska A, Tavaniello S, Żylińska J, Bardowski J, Bednarczyk M 2012: Influence of in ovo prebiotic and synbiotic administration on meat quality of broiler chickens. *Poultry Science* **91** 2963-2969.

Mansoub 2011: Growth performance and humoral immune response of broiler chicks fed diets containing graded levels of ground date pits with a mixture of dried neem, nishyinda and papaya and thyme. *Global Veterinarian* **6** 389-398.

Martin R, Olivares M, Marin ML, Fernandez L, Xaus J, Rodriguez JM 2005: Probiotic potential of 3 lactobacilli strains isolated from breast milk. *Journal of Human Lactation* **21(1)** 8-17.

Mastbaum I, Yossilewitsch L, Grimberg M, Kedem M, Viola S, Rand N, Dvorin A, Noy Y and Litman M 1997: Effects of the probiotic "Primalac" on broilers administered either as a feed additive or in the drinking water. In *11th European Symposium on Poultry Nutrition, Faaborg, Denmark* 511-513.

Min YN, HL Yang, YX Xu and YP Gao 2016: Effects of dietary supplementation of synbiotics on growth performance, intestinal morphology, sIgA content and antioxidant capacities of broilers. *Journal of Animal Physiology and Animal Nutrition* **100** 1073–1080.

Midilli, M, Kocabach M, Alp N, Muglah OH, Turan N, Yilmaz H and Cakir S 2008: Effects of dietary probiotic and prebiotic supplementation on growth performance and serum IgG concentration of broilers. *South African Journal of Science* **38** 21–27.

Moghadam AN, Pourreza J and Samie AH 2006: Effect of different levels of citric acid on calcium and phosphorus efficiencies in broiler chicks. *Pakistan Journal of Biological Science* **9** 1259-1256.

Mohan B, Kadireve R, Natarajan A, Bhaskaran M 1996: Effect of probiotic supplementation on growth, nitrogen utilization and serum cholesterol in broilers. *British Poultry Science* **37** 395-401.

Mohnl M, AcostaAragon Y, Acostaojedu A, Rodriguessanches B, Pasteiner S 2007: Effect of symbiotic feed additive in comparison to antibiotic growth promoter on performance and health status of broilers. *Poultry Science* **86** 217.

Mohyla P, Bilgili SF, Oyarzabal OA, Warf CC, Kemp GK 2007: Application of acidified sodium chlorite in the drinking water to control Salmonella serotype *Typhimurium* and *Campylobacter jejuni* in commercial broilers. *Journal of Applied Poultry Research* **16** 45-51.

Mountzouris KC, Tsirtsikos P, Kalamara E, Nitsch S, Schatzmayr G, Fegeros K 2007: Evaluation of the efficacy of a probiotics containing *Lactobacillus, Bacillus, Bifidobacterium, Enterococcus* and *Pediococcus* strains in promoting broiler performance and modulating cecal microflora composition and metabolic activities. *Journal of Poultry Science* **86** 309-317.

Mousavi SMA, Seidavi A, Dadashbeiki M, Kilonzo-nthenge A, Nahashon SN, Laudadio V, Tufarelli V, 2015: Effect of a synbiotic (Biomin ® IMBO) on growth performance traits of broiler chickens. European Poultry **79** 1-15

Muzaffar D, Fereda O, Kemal C 2003: Effect of dietary probiotic, organic acid and antibiotic supplementation to diets on broiler performance and carcass yield. *Pakistan Journal of Nutrition* **2** 89-91.

Nezhad YE, Shivazad M, Nazeeradl M, Babak MMS 2007: Influence of citric acid and microbial phytase on performance and phytate utilization in broiler chicks fed a corn-soybean meal diet. *Journal of the Faculty of Veterinary Medicine, University of Tehran* **61** 407-413.

Nisar H, Sharif M, Rahman MA, Rehman S, Kamboh AA, Saeed M 2020: Effects of dietary supplementations of synbiotics on growth

performance, carcass characteristics and nutrient digestibility of broiler chicken. *Brazilian Journal of Poultry Science* 1388.

Nahashon SN, Nakaue HS, Mirosh LW 1996: Performance of Single Comb White Leghorn fed a diet supplemented with a live microbial during the growth and egg laying phases. *Animal Feed Science and Technology* **57** 25–38.

Nourmohammadi R, Hosseini SM, Farhangfar H, Bashtani M 2012: Effect of citric acid and microbial phytase enzyme on digestibility of some nutrients in broiler chicks fed corn-soybean meal diets. *Italian Journal of Animal Science* **11(1)** 36–40.

Omar, Mohamed AE 2014: Economic evaluation of probiotic (*Lactobacillus acidophilus*) using in different broiler breeds within Egypt." *Benha Vet. Med. J* **26(2)** 52-60.

Panda AK, Raju MVLN, Rama Rao SV, Shyam Sunder G, Reddy MR 2009: Effect of graded levels of formic acid on gut microflora count, serum biochemical parameters, performance, and carcass yield of broiler chickens. *Indian Journal of Animal Science* **79** 1165-1168.

Parker RB 1974: Probiotics, the other half of the antibiotics story. *Animal Nutrition and Health* **29** 4–8.

Patterson JA, Burkholder KM 2003: Application of prebiotics and probiotics in poultry production. *The Journal of Poultry science* **82(4)** 627-31.

Patten JD and Waldroup PW 1988: Use of organic acids in broiler diets. *Poultry Science* **67** 1178-1182.

Paul RC, Ahmad N, Moinuddin MA, Hasan N 2010: Effects of administration of multivitamins and enzymes for broilers either singly or in combination on body weight and haemato- biochemical parameters. *Journal of Bangladesh Agricultural University* **8(1)** 39–44.

Philipsen IPLJ 2006: Acidifying drinking water supports performance. *World Poultry* **22** 20-21.

Pourakbari M, Seidavi A, Asadpour L and Martinez A 2014: Probiotic level effects on growth performance, carcass traits, blood parameters, cecal

microbita, and immune response of broilers. *Poultry Science* **93(12)** 97-103.

Rahimi S, Teymouri ZZ, Torshizi K, Omidbaigi R, Rokni H 2011: Effect of the three herbal extracts on growth performance, immune system, blood factors and intestinal selected bacterial population in broiler chickens. *Journal of Agricultural Science and Technology* **13** 527-539.

Raksasiri BV, Paengkoum P, Paengkoum S, Poonsuk K 2018: The effect of supplementation of synbiotic in broiler diets on production performance, intestinal histomorphology and carcass quality. *International Journal of Agricultural Technology* **14** 1743-1754.

Raka PS, Sjofjan O, Eka Radiati L 2014: Effect of Liquid Probiotics Mixed Culture Supplements through Drinking Water on Laying Hens Performance and Yolk Cholesterol. *Journal of World's Poultry Research* **4** 05-09.

Roy BC and Chowdhury SD 2013: Effect of dietary probiotic and antibiotic growth promoter either alone or in combination on the growth performance of broilers during summer. *Eighth International Poultry Show and Seminar, WPSA-BB* 153-158.

Saadia M, Nagla KS 2010: Effect of probiotic (*Saccharomyces cerevisiae*) adding to diets on intestinal microflora and performance of Hy-Line layers hens. *Journal of American Science* **6** 159-169.

Samanta M, Biswas P 1995: Effect of feeding probiotic and lactic acid on the performance of broiler. *International Journal of Poultry Science* **30** 145-147.

Samanta S, Haldar S, Ghosh TK. 2010: Comparative efficacy of an organic acid blend and bacitracin methylene disalicylate as growth promoters in broiler chickens: effects on performance, gut histology and small intestinal milieu. *Veterinary Medicine International* **8** ID 645150.

Sarangi NR, Babu LK, Kumar A, Pradhan CR, Pati PK and Mishra JP 2016: Effect of dietary supplementation of prebiotic, probiotic, and synbiotic on growth performance and carcass characteristics of broiler chickens. *Veterinary World* **9** 313–319.

Shokri AN, Ghasemi HA and Taherpour K (2017): Evaluation of Aloe vera and symbiotic as antibiotic growth promoter substitutions on performance, gut morphology, immune responses and blood constitutes of broiler chickens. *Journal of Animal Science* **88** 306–313.

Shen HF, Han CW and Du-Bing W 2005: Effect of citric acid on production performance of three yellow chickens. *China Poultry* **27** 14-15.

Shim YH, Ingali SL, Kim JS, Seo DK, Lee SC and Kwon IK 2012: A multi microbe probiotic formulation processed at low and high drying temperatures: effects on growth performance, nutrient retention and caecal microbiology of broilers. *Journal of British Poultry Science* **53** 482-490.

Singh S, Sharma VP, Singh S 1999: Performance of broiler chicks under different energy and probiotic levels during summer season. *Indian Journal of Poultry Science* **34** 34-37.

Sohail MU, Hume ME, Byrd JA, Nisbet DJ, Shabbir MZ, Ijaz A, Rehman H 2015: Molecular analysis of the caecal and tracheal microbiome of heat stressed broilers supplemented with prebiotic and probiotic. *Avian Pathology* **44** 67-74.

Stavric S, Komegay ET 2008: 10 Microbial probiotics for pigs and poultry. *Biotechnology in Animal Feeds and Animal Feeding* 205.

Suparom W, Chumpawadee S, Santaweesuk N, Khajarern J 2013: Efficacy of symbiotic in broiler die. *Khon Kaen Agricultural Journal* **41** 381-387.

Tayeri V, Seidavi A, Asadpour L, Phillips CJC 2018: A comparison of the effects of antibiotics, probiotics, synbiotics and prebiotics on the performance and carcass characteristics of broilers. *Veterinary Research Communications* **42** 195–207.

Thompson JL and Hinton M 1997: antibacterial activity of formic and propionic acids in the diet of hens on salmonellas in the crop. *British Poultry science* **38** 59-65.

Timmerman HM, Veldman A, Elsen E, Rombouts F, Beynen AC 2006: Mortality and Growth Performance of Broilers Given Drinking Water

Supplemented with Chicken-Specific Probiotics. *Poultry Science* **85** 1383-8.

Torshizi MA, Moghaddam AR, Rahimi SH, Mojgani N 2010: Assessing the effect of administering probiotics in water or as a feed supplement on broiler performance and immune response. *British Poultry Science* **51** 178-184.

Tortuero F 1973: Influence of the implantation of Lactobacillus acidophilus in chicks on the growth, feed conversion, malabsorption of fats syndrome and intestinal flora. *Poultry Science* **52(1)** 197-203.

Tufan T and Bolacali M 2017: Effects of dietary addition of synbiotic on the performance, carcass traits, and serum parameters of Japanese quails. *Revista Brasileira de Zootecnia* **46(10)** 805-813.

Yang Y, Iji PA, Choct M 2009: Dietary modulation of gut micro flora in broiler chickens: a review of the role of six kinds of alternatives to in-feed antibiotics. *World's Poultry Science Journal* **65** 97-114.

Verma SVS (1992): Prospects of improving nutritive value of poultry feeds through biotechnology. *Poultry Guide* **29** 37-39.

WHO (World Health Organization) 1997: The medical impact of use of antimicrobials in food animals. Report of a WHO Meeting. Berlin, Germany.

Wondwesen A, Moges S 2017: Review on application of probiotics in poultry production. *British Journal of Poultry Science* **6** 46-52.

Zhang L, Zhang R, Jia H, Zhu Z, Li H, Ma Y 2021: Supplementation of probiotics in water beneficial to growth performance, carcass traits, immune function and antioxidant capacity in broiler chickens. *Open Life Sciences* **16** 311-322.

Zhang ZF, Kim IH 2014: Effects of multistrain probiotics on growth performance, apparent ileal nutrient digestibility, blood characteristics, cecal microbial shedding, and excreta odor contents in broilers. *Poultry Science* **93(2)** 364-70.

Zhang AW, Lee DB, Lee SK, Lee KW, An GH, Song KB and Lee CH 2005: Effects of yeast (*saccharomyces cerevisiae*) cell components on growth performance, meat quality and ileal mucosa development of broiler chicks. *Poultry Science Association, Inc.* ***84***(7), 1015-1021

Zhang G, Ma L, Doyle MP 2006: Efficiency of probiotics, prebiotics and synbiotics on weight increase of chickens. (*Gallus Domesticus*).

Ziggers D 2011: Animal Feed News. EU 12-point antibiotic action plan released, 18 November, 2011.

APPENDICES

Appendix 1: Body weight (g/bird) of broilers in different dietary treatments at different ages

Treatment	Replication	Age (week)					
		Day old	1st	2nd	3rd	4th	5th
Control	R_1	44.50	206.48	537.23	1058.26	1482.12	1740.15
	R_2	45.90	210.64	553.10	1060.15	1480.32	1743.09
	R3	46.60	201.12	535.11	1070.98	1485.75	1740.23
	R_4	45.80	208.88	539.15	1060.45	1482.82	1741.20
Average		45.70	206.78	541.15	1062.46	1482.76	1741.17
Citric acid	R_1	46.10	208.75	538.15	1054.21	1495.85	1773.75
	R_2	44.50	210.62	545.98	1067.87	1505.95	1789.82
	R_3	42.60	215.26	561.80	1021.32	1488.15	1750.17
	R_4	44.00	211.50	548.74	1050.47	1496.64	1770.25
Average		44.30	211.53	548.67	1051.47	1496.65	1771.00
Synbiotic	R_1	47.60	215.23	550.63	1048.25	1496.65	1774.05
	R_2	43.80	206.86	556.15	1053.20	1506.23	1792.85
	R_3	41.60	207.74	549.22	1040.45	1517.23	1753.15
	R_4	45.40	210.48	538.68	1047.30	1488.13	1770.68
Average		44.60	209.58	548.67	1047.32	1503.86	1772.68
Probiotic	R_1	43.50	211.82	556.31	1060.23	1538.15	1790.13
	R_2	45.10	214.41	550.15	1072.88	1522.25	1783.17
	R_3	47.90	210.45	567.21	1065.92	1542.14	1813.23
	R_4	47.50	213.59	531.25	1079.68	1531.53	1792.11
Average		45.50	212.57	551.23	1069.68	1533.52	1782.16

Appendix 2: Body weight gain (g/bird) of broilers in different dietary treatments at different ages

Treatment	Replication	Age (week)				
		1st	2nd	3rd	4th	5th
Control	R_1	162.78	330.75	519.23	425.87	258.03
	R_2	164.94	342.46	507.03	420.11	262.80
	R_3	156.53	331.89	537.87	414.87	254.38
	R_4	160.07	332.37	521.52	420.35	258.42
**Average**		161.08	334.37	521.3	420.3	258.41
Citric acid	R_1	162.45	329.4	516.11	441.64	277.9
	R_2	165.32	335.36	533.89	446.08	283.88
	R_3	172.92	347.63	458.45	467.83	260.02
	R_4	168.23	336.16	502.85	425.18	276.59
**Average**		167.23	337.14	502.8	445.18	274.60
Synbiotic	R_1	167.63	335.39	497.62	457.98	277.83
	R_2	162.16	349.39	497.11	463.97	273.62
	R_3	165.12	342.49	511.22	447.71	265.02
	R_4	164.96	339.10	498.65	456.54	258.81
**Average**		164.98	329.09	488.65	456.57	268.82
Probiotic	R_1	169.32	343.79	503.92	477.92	251.98
	R_2	170.91	336.72	522.72	449.37	260.86
	R_3	162.95	336.78	518.69	462.22	273.08
	R_4	165.09	337.36	528.46	465.85	248.64
		167.07	338.66	518.45	463.84	258.65

Appendix 4: Feed intake (g/bird) of broiler in different dietary treatments at different ages

Treatment	Replication	Age (week)				
		1st	2nd	3rd	4th	5th
Control	R_1	166.75	533.13	764.15	832.01	676.5
	R_2	167.94	546.75	767.14	829.54	688.12
	R_3	166.53	532.15	772.51	827.55	680.5
	R_4	168.80	537.34	767.933	829.7	681.71
**Average**		167.5	537.34	767.933	829.7	681.71
Citric acid	R_1	171.59	527.35	769.25	827.54	623.15
	R_2	172.53	535.84	771.52	823.52	639.5
	R_3	167.58	542.15	770.54	828.51	632.75
	R_4	170.57	535.113	770.437	826.52	631.8
**Average**		170.57	535.113	770.437	826.52	631.8
Synbiotic	R_1	164.77	530.12	768.52	827.76	612.11
	R_2	165.60	539.55	771.75	828.30	618.50
	R_3	168.66	538.11	779.16	829.15	627.75
	R_4	165.60	535.98	761.15	828.20	618.15
**Average**		166.65	535.94	770.14	828.35	619.13
Probiotic	R_1	159.05	537.17	771.15	826.58	598.23
	R_2	165.23	534.15	771.14	828.52	618.60
	R_3	162.10	532.04	762.21	829.95	621.13
	R_4	161.05	533.13	781.5	826.08	619.20
**Average**		162.06	534.12	771.5	827.78	609.29

Appendix 4: FCR of broilers in different dietary treatments at different ages

Treatment	Replication	Age (week)				
		1st	2nd	3rd	4th	5th
Control	R_1	1.05	1.61	1.46	1.96	2.62
	R_2	1.06	1.59	1.51	1.97	2.61
	R3	0.98	1.60	1.43	1.99	2.67
	R_4	1.05	1.63	1.47	1.95	2.66
Average		1.04	1.61	1.47	1.97	2.64
Citric acid	R_1	1.03	1.60	1.47	1.97	2.43
	R_2	1.00	1.60	1.49	1.87	2.24
	R_3	1.03	1.59	1.47	1.87	2.25
	R_4	1.04	1.56	1.67	1.77	2.41
Average		1.02	1.59	1.54	1.85	2.30
Synbiotic	R_1	1.01	1.59	1.54	1.84	2.30
	R_2	1.02	1.58	1.54	1.80	2.28
	R_3	0.97	1.56	1.53	1.78	2.24
	R_4	1.04	1.57	1.56	1.85	2.36
Average		1.01	1.58	1.54	1.81	2.30
Probiotic	R_1	0.96	1.58	1.46	1.81	2.25
	R_2	0.94	1.56	1.53	1.72	2.27
	R_3	1.01	1.59	1.47	1.84	2.23
	R_4	1.00	1.57	1.54	1.73	2.27
Average		0.97	1.58	1.49	1.78	2.25

Appendix 5: Blood biochemical parameters of broiler in different dietary treatments (mg/dl)

Treatment	Replication	Cholesterol (mg/dl)	TG (mg/dl)	HDL (mg/dl)	LDL (mg/dl)
Control	R_1	102.32	61.22	38.98	51.09
	R_2	110.62	66.30	38.06	60.89
	R_3	122.92	69.38	35.15	73.89
	R_4	114.62	64.3	36.06	64.62
**Average**		112.62	65.3	37.06	62.62
Citric acid	R_1	143.52	60.4	41.32	90.12
	R_2	79.06	74.28	34.2	30
	R_3	101.27	77.64	28.78	40.16
	R_4	121.31	57.04	46.74	80.01
**Average**		111.29	67.34	37.76	60.06
Synbiotic	R_1	109.96	78.95	35.27	60.14
	R_2	106.31	67.75	37.05	55.71
	R_3	111.62	88.16	37.52	56.46
	R_4	107.96	76.95	39.29	52.02
**Average**		108.96	77.95	37.28	56.08
Probiotic	R_1	128.23	72.65	33.72	79.98
	R_2	148.83	72.02	31.49	94.95
	R_3	169.43	91.42	33.25	117.89
	R_4	148.83	92.04	35.47	102.91
**Average**		148.83	82.03	33.48	98.93

Appendix 6: Dressing parameters and meat and bone development of broilers in different dietary treatments (% in relation to body weight)

Treatment	Replication	Live wt (g)	Bone meat (g)	Thigh meat wt (g)	Thigh bone wt (g)	Drumstick meat wt (g)	Drumstick bone wt (g)	Wing meat wt (g)	Wing bone wt (g)	Head wt (g)	Neck wt (g)	Leg wt (g)	Liver wt (g)	Heart wt (g)	Gizzard wt (g)	Abdominal fat wt (g)
Control	R_1	1620	300	140	28	96	44	56	44	22	32	32	40	8	26	10
	R_2	1660	340	150	34	108	48	58	48	22	38	32	42	11	27	8
	R_3	1780	380	160	40	120	52	60	52	22	44	32	44	14	28	6
	R_4	1687	340	150	34	108	48	58	48	22	38	32	42	11	27	8
Citric acid	R_1	1690	292	128	36	88	44	52	44	14	34	36	46	10	30	6
	R_2	1840	340	132	36	108	52	60	48	16	38	42	38	12	30	4
	R_3	1765	316	130	36	98	48	56	46	15	36	39	42	11	30	5
	R_4	1765	316	130	36	98	48	56	46	15	36	39	42	11	30	5
Synbiotic	R_1	1810	332	136	35	96	52	60	40	20	36	36	42	9	27	6
	R_2	1760	344	128	36	96	56	60	36	18	34	38	44	8	26	8
	R_3	1860	320	144	36	96	48	60	44	22	38	34	40	10	28	4
	R_4	1810	332	136	36	96	52	60	40	20	36	36	42	9	27	6
Probiotic	R_1	1726	372	124	28	92	48	56	44	18	26	40	36	8	30	6
	R_2	1758	354	142	34	106	50	56	54	18	34	43	35	9	30	5
	R_3	1790	336	160	40	120	52	56	64	18	42	46	34	10	30	4
	R_4	1758	354	142	34	106	50	56	54	18	34	43	35	9	30	5
